# Genomic Analysis Reveals Novel Diversity among the 1976 Philadelphia Legionnaires’ Disease Outbreak Isolates and Additional ST36 Strains

**DOI:** 10.1371/journal.pone.0164074

**Published:** 2016-09-29

**Authors:** Jeffrey W. Mercante, Shatavia S. Morrison, Heta P. Desai, Brian H. Raphael, Jonas M. Winchell

**Affiliations:** Pneumonia Response and Surveillance Laboratory, Respiratory Diseases Branch, Centers for Disease Control and Prevention, Atlanta, Georgia, United States of America; University of Louisville, UNITED STATES

## Abstract

*Legionella pneumophila* was first recognized as a cause of severe and potentially fatal pneumonia during a large-scale outbreak of Legionnaires’ disease (LD) at a Pennsylvania veterans’ convention in Philadelphia, 1976. The ensuing investigation and recovery of four clinical isolates launched the fields of *Legionella* epidemiology and scientific research. Only one of the original isolates, “Philadelphia-1”, has been widely distributed or extensively studied. Here we describe the whole-genome sequencing (WGS), complete assembly, and comparative analysis of all Philadelphia LD strains recovered from that investigation, along with *L*. *pneumophila* isolates sharing the Philadelphia sequence type (ST36). Analyses revealed that the 1976 outbreak was due to multiple serogroup 1 strains within the same genetic lineage, differentiated by an actively mobilized, self-replicating episome that is shared with *L*. *pneumophila* str. Paris, and two large, horizontally-transferred genomic loci, among other polymorphisms. We also found a completely unassociated ST36 strain that displayed remarkable genetic similarity to the historical Philadelphia isolates. This similar strain implies the presence of a potential clonal population, and suggests important implications may exist for considering epidemiological context when interpreting phylogenetic relationships among outbreak-associated isolates. Additional extensive archival research identified the Philadelphia isolate associated with a non-Legionnaire case of “Broad Street pneumonia”, and provided new historical and genetic insights into the 1976 epidemic. This retrospective analysis has underscored the utility of fully-assembled WGS data for *Legionella* outbreak investigations, highlighting the increased resolution that comes from long-read sequencing and a sequence type-matched genomic data set.

## Introduction

In the summer of 1976, an estimated 4400 American Legionnaires, Legion Auxiliary, and guests gathered at a downtown Philadelphia hotel on Broad Street (“hotel A”) for the 58^th^ Annual Convention of the American Legion, Department of Pennsylvania [1, 2]. Shortly after the close of ceremonies, the Centers for Disease Control (CDC) in Atlanta received a call describing a cluster of patients with severe febrile respiratory illness who recently attended this convention, some of whom died. In the months to follow, numerous health officials were deployed to investigate this epidemic [3, 4]. Two hundred twenty-one individuals ultimately met the clinical criteria for the respiratory syndrome known as Legionnaires’ disease (LD); 34 people succumbed to their infections [1].

After numerous attempts at cultivation with various procedures, scientists at the CDC recovered a previously unknown bacterium from autopsy lung tissue of three cases associated with this unprecedented outbreak, and also from one bystander who did not enter the convention hotel (designated “Broad Street pneumonia”) [5]. These four strains became the foundation for the epidemiological, environmental, and laboratory study of the genus *Legionella*, that now encompasses ~60 species [6]. Data collected during this and subsequent LD epidemics, as well as reexaminations of past outbreaks, helped classify *Legionella pneumophila* as an agent of severe and potentially deadly pneumonia that could be spread from seemingly innocuous sources [7–11].

The initial LD isolate, Philadelphia-1, became the *L*. *pneumophila* serogroup 1 (sg1) type strain that was widely distributed and studied, and is among the first *Legionella* strains to be entirely sequenced [12]. Our understanding of its relationship to the remaining isolates, Philadelphia-2, -3, and -4, comes from an extensive epidemiological investigation suggesting a common source of infection [1] and from a handful of early laboratory studies [13–17]. Further analysis of these 3 isolates is lacking in part because they are not commercially available and no genome sequence information exists. Thus, their exact molecular and epidemiological relationships to the Philadelphia-1 isolate remain unexplored until now.

As part of the CDC Advanced Molecular Detection initiative, which supports the development of genetic characterization methods for next-generation *Legionella* detection and typing, the present study leveraged innovative sequencing technologies and bioinformatics capabilities to retrospectively investigate the first *Legionella* outbreak from which multiple clinical isolates are available. Through in-depth historical research we also sought to more fully describe the clinical cases from which these isolates were recovered, with emphasis toward the unidentified case of “Broad Street pneumonia”. Using a background of environmental and clinical isolates sharing the strain Philadelphia sequence type (ST36) collected over 30 years, we resolved the genetic relationships among these historical Philadelphia strains. Further, we characterized genomic features that may promote pathogenicity, and described high-density polymorphic regions that present opportunities for targeted genetic assay development that potentially could be useful during *Legionella* outbreak investigations.

## Materials and Methods

### Growth and Culture of *Legionella*

The earliest *L*. *pneumophila* “Philadelphia” strains in the CDC archival collection, dating from 1977 and 1978, were chosen for the current study, along with more recent isolates sharing the Philadelphia sequence type (ST36), to provide a relevant background for phylogenetic comparison. These ST36 comparison strains were selected from the CDC *Legionella* stock collection to be geographically and temporally diverse, as well as to represent both clinical and environmental settings in outbreak investigations, sporadic cases, and unassociated with disease. Strains originated from 15 U.S. states and were isolated in the period from 1982 to 2013. Historical Philadelphia strains in the CDC collection were originally stored as pure culture stocks at -80°C in rabbit blood within vacuum-sealed glass ampules. Archival stocks of strains Philadelphia-1 and -2 were stored on November 17^th^, 1977, while strains Philadelphia-3 and -4 were stored on April 10^th^, 1978. These stocks were aseptically plated directly from the original frozen stocks onto solid BCYE media containing L-cysteine inside a biological safety cabinet using a sterile 10ul loop. Plated cultures were incubated for 3–5 days at 35°C in a humidified, 2.5% CO_2_ atmosphere as described previously [18–20]. Isolated single colonies were chosen from these initial platings, examined by low power microscopy, tested for cysteine auxotrophy on BCYE bi-plates, plated for growth on solid BCYE, and then processed as described below.

### Genomic DNA Extraction and NGS Library Preparation

Genomic DNA (gDNA) was extracted from pure *Legionella* cultures using the Epicentre Masterpure DNA Purification Kit (cat. no. MCD85201, Epicentre, Madison, WI), as per the manufacturer’s instructions. Briefly, approximately half of a sterile 10ul loop of each pure strain was taken from a BCYE plate after 3 days of growth, suspended in 300ul of Tissue and Cell Lysis Solution with 1ul of Proteinase K (initial concentration of 50ug/ul), and incubated at 65°C for 15 minutes with occasional vortexing. Samples were cooled, 1ul of RNaseA (initial concentration of 5ug/ul) was added and tubes were incubated at 37°C for 30 minutes. Cell lysates were then placed on ice for 5 minutes, 175ul of MPC Protein Precipitation Reagent was added, samples were vortexed for 10 seconds and then centrifuged at 14,000 x g for 10 minutes. The clarified cell extract was removed, combined with 500ul of isopropanol, inverted 40 times and centrifuged at 16,000 x g for 10 minutes. The supernatant was then discarded and the gDNA pellet was washed twice with 1.5ml of 70% ethanol, air dried for 5 minutes and suspended in 50ul of Tris-HCl buffer, pH 8.0. DNA concentration was measured by the Qubit Fluorometric Quantitation system (Life Technologies, Carlsbad, CA) using either the dsDNA broad range (cat. no. Q32853) or high sensitivity (cat. no. Q32851) assay kit. Typical gDNA yield was between 10-50ug.

In preparation for Illumina MiSeq sequencing, 2 μg of gDNA was sheared to an average fragment length of 600 bp using a Covaris M220 ultrasonicator (Woburn, MA), cleaned and concentrated with Agencourt AMPure XP magnetic beads (cat. no. A63880, Beckman Coulter, Indianapolis, IN) as per the manufacturer’s instructions with one minor modification (80% ethanol used for both washes), and eluted in Tris-HCl, pH 8.0. DNA quality (260:280 and 260:230) was measured on a Nanodrop ND-1000 spectrophotometer (Thermo Scientific, Wilmington, DE) and initial average fragment size was verified on the Agilent Tapestation 2200 (Santa Clara, CA) using the D1000 ScreenTape and reagents (cat. no. 5067–5582 and 5067–5583) with an external ladder. Illumina-compatible libraries were constructed with 1ug of sheared input DNA on a Zephyr Molecular Biology Workstation (Perkin Elmer, Waltham, MA) using custom scripts written to follow the manufacturer’s protocol for the NEBNext Ultra DNA Library Preparation Kit (cat. no. E7370, New England Biolabs, Ipswich, MA) combined with NEBNext Multiplex Oligo Index Primer Sets 1 and 2 (cat. no. E7335 and E7500). NEB USER enzyme was included during the PCR cycling step, with a subsequent AMPure magnetic bead cleanup (at 0.7X) and elution into Tris-HCl, pH 8.0. Indexed libraries were normalized, pooled, denatured, and diluted as per Illumina sequencing instructions (“Preparing Libraries for Sequencing on the MiSeq” Part# 15039740 Rev. D), and loaded onto a MiSeq v2 Reagent Kit (cat. no. MS-102-2003) for 2 x 250 bp paired-end sequencing.

Library preparation for Pacific Biosciences (PacBio) RSII long-read sequencing (Menlo Park, CA) began by shearing 8 ug of purified gDNA to an average size of 15,000 bp using a Covaris g-tube (cat. no. 520079) as per the manufacturer’s instructions, followed by cleaning and concentration with pre-washed AMPure XP magnetic beads and suspension in Pacific Biosciences Elution Buffer. Average sheared fragment size was verified on an Agilent Tapestation 2200 using the Genomic DNA ScreenTape and reagents (cat. no. 5067–5365 and 5067–5366) and quality parameters were measured by a Nanodrop ND-1000 spectrophotometer. PacBio-compatible libraries were constructed with 5 ug of sheared input DNA using the SMRTbell Damage Repair Kit (PN 100-465-900) and SMRTbell Template Prep Kit 1.0 (PN 100-259-100) together with AMPure PB magnetic beads (PN 100-265-900) on a Zephyr Molecular Biology Workstation using custom scripts that follow the PacBio 10-kb Template Preparation and Sequencing protocol (PN 100-092-800-06). Final average PacBio library size was measured on the Agilent Tapestation 2200 using the Genomic DNA ScreenTape. In preparation for sequencer loading, the PacBio Binding Calculator v.2.3.1.1 was used to construct a manual protocol for primer annealing, DNA polymerase binding, and MagBead binding using the DNA Polymerase Binding Kit P6 v2 (PN 100-372-700) and MagBead Kit (PN 100–133600). PacBio RSII sequencing runs were performed with a 2-kb DNA Internal Control Complex (PN 100-356-500), 240 minute movie time, and stage start using DNA Sequencing Reagent Bundle 4.0 (PN 100-356-400) at 1 or more SMRT Cells per prepared library.

### Whole Genome Assembly

The Hierarchical Genome Assembly Process version 3 (HGAP3) was used to construct the complete *L*. *pneumophila* genome sequences [21]. The HGAP3 expected genome size and target genome coverage parameters were set to 3.4 Mb and 15X, respectively. The minimum subread length value was adjusted to decrease the genome coverage to the recommended 100 -150X for microbial genomes[22]. Genome closure was performed by identifying and trimming nucleotide overlap at the ends of the single assembled contig sequences with Gepard v.1.3 [23]. The reformatted genome sequence was used as input for the RS-ReSequencing protocol within the PB SMRT analysis portal to construct the polished genome sequence for each isolate. Optical maps were generated for several isolates for comparison with the polished genomic sequence to identify any potential misassembles and to ensure that the HGAP assembly generated the correct genome structure. A single optical map disagreement was resolved with the RS-BridgeMapper protocol within the SMRT analysis portal. We aligned paired-end Illumina read data for each sequenced isolate to its respective PacBio polished sequence using Bowtie v.2.1.0 [24]. Any nucleotide discrepancies between the two data were identified with Samtools v.0.1.18 [25] and FreeBayes v.0.9.21 [26] (http://arxiv.org/abs/1207.3907v2) and the Illumina data set was used as the final determinate for the nucleotide sequence. VCFtools v.0.1.11 was used to construct the final consensus sequence using both data types [27]. Final complete PacBio genome assemblies and raw Illumina sequencing reads for genomes sequenced in the present study were deposited at NCBI under BioProject PRJNA323476. All Illumina sequencing data was assigned the SRA accession SRP075902, and individual *L*. *pneumophila* strains were given PacBio complete genome/Illumina SRA accession identifiers as described in S1 Table.

### Gene Prediction and Whole-Genome Comparison with *L*. *pneumophila* str. Philadelphia-1

All fully assembled genome sequences were re-oriented beginning at the origin of replication to ensure that the first gene predicted was *dna*A. Prodigal v.2.6 [2] was used to predict the amino acid coding sequences for each newly sequenced *L*. *pneumophila* isolate, and the NCBI *L*. *pneumophila* str. Philadelphia-1 (NC_002942) [12] reference sequence was used in comparative genome analyses. Whole-genome alignments were performed and visualized (from pairwise LCBs) using the Mauve progressive algorithm [28] or MAFFT v.7.215 [29]. On two occasions, at nucleotide positions ~350,000 and ~2,651,000 relative to the NCBI strain Philadelphia-1 reference, the MAFFT multiple sequence alignment was manually edited using the Geneious v.8.1 software suite (Biomatters, Ltd., New Zealand) to correct obvious misalignment. Orthologous protein relationships were defined with PanOct v.1.9 based on amino acid sequences sharing sequence identity of ≥55% and coverage of ≥60%, as described previously [30]. Phylogenetic and in-depth gene content analyses were performed with the pan-genome dataset generated from the PanOct analysis. The BLAST ring image generator (BRIG) was used to visually compare genome content relative to *L*. *pneumophila* str. Philadelphia-4 [31]. Additional *Legionella* genomes from the NCBI genome repository used in the present study included *L*. *pneumophila* strains Paris (NC_006368), ATCC 43290 (NC_016811; sg12), Lorraine (NC_018139), LPE509 (NC_020521), 130b (NZ_CAFM00000000), Corby (NC_009494), Alcoy (NC_014125), and *L*. *longbeachae* NSW150 (NC_013861).

### Phylogenetic Analyses

Phylogenetic trees were constructed using both core-protein coding ORFs and a DNA core-SNP-based approach. Multiple sequence alignment for the core-gene analysis was performed according to Morrison *et al*. [32], except that Clustal Omega v.1.2 was used in place of ClustalW [33]. RAxML v.8, a maximum-likelihood algorithm, was used to generate the phylogenetic tree with the GTRGAMMA substitution model and 1,000 bootstrappings [34] for both analyses. The core-SNP-based phylogenetic comparison was generated with kSNP v.3 [35]. Genomic loci potentially subject to recombination were identified using Gubbins v.1.4.1 [36] and a custom script was used to mask these regions within a MAFFT multiple sequence alignment file. kSNP was then used for the identification of core, non-recombining SNPs within these masked sequences, and a maximum-likelihood tree was generated with RAxML.

### Genome and Gene Content Visualization

Gene content visualizations and trees were created using the interactive tree of life (iTOL) (http://itol.embl.de/) [37], Geneious v.8.1.7, and InkScape v.0.48.5 (https://inkscape.org/en/).

## Results

### Archival Research and Original LD Case Descriptions

An extensive examination of the published literature and unpublished primary records associated with the 1976 Philadelphia outbreak was conducted at the CDC in Atlanta and one additional repository. Recovered documents included specimen accession logs, laboratory experimental results, field notes, and correspondence between scientists and public health officials involved in the original investigation. Records reflect that early microscopic examinations and efforts at cultivating the LD agent yielded only presumed bacterial contaminants. In January 1977, CDC scientists successfully isolated the Legionnaires’ disease agent from among these “contaminants” using classic rickettsial laboratory techniques [5, 38]. This previously unknown, acid fast, Gram negative bacillus was recovered from the lung tissues of 4 separate pneumonia cases, and subsequently given the strain designations Philadelphia-1, -2, -3 and -4. Upon recent propagation from archival frozen culture stocks (see Methods), CDC *L*. *pneumophila* strains Philadelphia-1, -2 and -4 displayed typical *Legionella* growth and colony morphology, including a “cut/ground glass” appearance and entire margin with iridescence. Strain Philadelphia-3 exhibited similar growth kinetics, but after 4 days of incubation all colonies formed an opaque “cap” encircled by translucent growth. The basis for this phenotype is unknown.

The four cases from which legionellae were recovered (Table 1) shared many risk factors with the larger infected Legionnaire population and frequently described in “at-risk” groups, including cigarette smoking, obesity, alcoholism, diabetes, emphysema, and heart problems [1]. All 4 patients were Caucasian, 3 were male, but only 2 were Legion delegates, and the single female case (strain Philadelphia-3) was the wife of a Legionnaire. Of significant epidemiological importance is the isolate designated “Philadelphia-1”, which originated from one of 39 identified cases of Broad Street pneumonia. This individual was reportedly exposed on a single day (July 23^rd^, 1976) in a defined time period as he stood on the sidewalk in front of hotel A, in part to watch the convention parade. As with all cases of Broad Street pneumonia, he never entered the implicated hotel. Interestingly, while the cases associated with Philadelphia strains -2, -3, and -4 originally reported multiple potential exposures at hotel A, they all resided at a different hotel (“hotel E”) located 1/3^rd^ of a mile from the official convention headquarters on Broad Street.

**Table 1:** Description of cases from whom *Legionella* was isolated during the 1976 Legionnaires' disease outbreak in Philadelphia.

Original Isolate Designation	Final Strain Designation	Original Isolation Date	Source of Isolate	Age	Sex	Ethnicity	Occupation	Convention Affiliation	Potential Exposure	Hotel of Residence	Pre-existing Conditions	Peak Temperature (°F) During Illness	Date of Disease Onset	Date of Death and Sample Collection	Potential Exposure to Disease Onset (Days)
Legionnaires' Isolate #1	Philadelphia-1	1/11/1977	Autopsy lung tissue	47	Male	Caucasian	Bus driver	Broad Street Pneumonia	Single day exposure—stood on the sidewalk in front of hotel A to watch parade on 7/23	None	Obesity and light smoker	105.2	7/28/1976	8/2/1976	5
Legionnaires' Isolate #2	Philadelphia-2	1/11/1977	Autopsy lung tissue	41	Male	Caucasian	Publishing consultant/salesman	Legionnaire—Delegate	Attended one or more Legion meetings from 7/21-7/24	Hotel E	Alcoholism, diabetes, obesity, moderate smoker	105	7/26/1976	8/2/1976	2–5
Legionnaires' Isolate #3	Philadelphia-3	1/16/1977	Autopsy lung tissue	55	Female	Caucasian	Retired hospital laboratory staff	Wife of Legionnaire	Waited in the lobby of Hotel A on more than one occasion, 7/21-7/24	Hotel E	History of myocardial infarction and atrial and ventricular tachycardia, heavy smoker, high blood pressure, hyperlipidemia, chronic cough	105.4	7/24/1976	8/4/1976	0–3
Legionnaires' Isolate #4	Philadelphia-4	1/16/1977	Autopsy lung tissue	63	Male	Caucasian	Retired machinist/coal miner	Legionnaire—Delegate	Attended one or more Legion meetings, hospitality rooms, and parade from 7/21-7/24	Hotel E	Emphysema, moderate smoker	105	7/25/1976	8/5/1976	1–4

### Sequencing and Shared Gene Content

**Fig 1:**
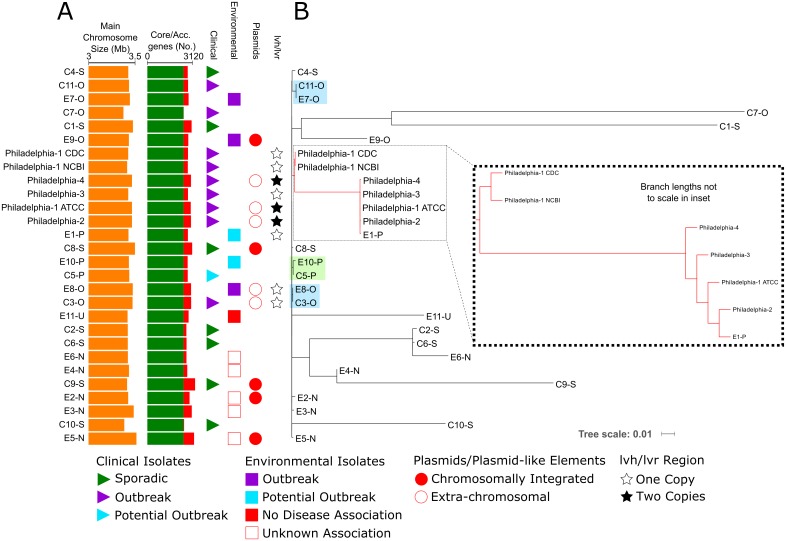
Genomic characteristics and core-SNP-based phylogenetic analyses of all *L*. *pneumophila* strains sequenced in the present study. **(A)** Genomic characteristics of all sequenced strains are shown as orange bars, green bars, and red stacked bars representing genome size, core genes (2828 genes), and accessory genes outside of the core, respectively. **(B)** Maximum-likelihood tree based on 11,356 core SNPs identified in all genomes. The Philadelphia clade is outlined and also expanded, therefore branches are not to scale in the inset. Blue and green shaded boxes highlight the confirmed (-O), and potential (-P) outbreak isolate pairs, respectively. Units of branch length (“Tree Scale”) are in nucleotide substitutions per site.

Twenty-seven *L*. *pneumophila* sg1 isolates were chosen for Pacific Biosciences (PacBio) long-read and Illumina long-insert, paired-end sequencing (S1 Table), including the 4 original 1976 Philadelphia isolates, the American Type Culture Collection Philadelphia-1 isolate (ATCC 33152), and 22 additional ST36 strains. All genomes assembled into single main, circular chromosomes between 3,336,955 and 3,465,362 bp, with smaller, circular extrachromosomal elements assembled in 5 strains that ranged from 48,354 to 73,576 bp (Fig 1A and S1 Table). Notably, the same plasmid (pCIN2) was shared between strains E5-N and C9-S. The number of common, core genes predicted among the entire 27 genome collection (2828 genes), plus the NCBI *L*. *pneumophila* str. Philadelphia-1 reference sequence (NC_002942), was generally consistent with previous reports[30, 39, 40], and this core gene subset represented ~93% of the gene content found in all Philadelphia strains. The accessory gene complement averaged ~210 (SD ± 33.7) (Fig 1A), but varied between 142 and 286 genes. The clinical isolate genome subset (n = 17), which also included all presently sequenced Philadelphia strains plus the NCBI reference sequence, contained 266 genes unique to a least one member of this group, while the environmental subset (n = 11) contained 145 genes not found in the clinical strains. The Philadelphia strains contributed 44 novel genes to the clinical subset, most of which were concentrated in the first ~210,000 bp of each genome (data not shown). The unique clinical and environmental genes identified will be discussed in a subsequent report.

### Phylogenetic Analyses

Initial *Legionella* research conducted in the 1970’s and ‘80’s indicated that the Philadelphia LD isolates were indistinguishable in antibody reactivity [15, 16], enzyme complement [13], and nucleotide complementarity [17]. Therefore, we examined their presumed genetic relationships by both core-SNP and core-gene phylogenetic approaches [32, 41], with the additional ST36 genomes as a relevant genetic background (Fig 1B and S1 Fig). One potential (C5-P/E10-P) and two confirmed (C3-O/E8-O and C11-O/E7-O) outbreak-associated clinical/environmental isolate pairs were included in the dataset to validate that these methods could cluster isolates from the same epidemiologically-linked LD events. Both phylogenetic analyses assigned all historical Philadelphia sequences to a single clade with two distinct branches (Fig 1B inset, and S1 Fig). One branch included the CDC and NCBI strain Philadelphia-1 sequences, and the other encompassed strains Philadelphia-2, -3, -4, ATCC Philadelphia-1, and environmental isolate E1-P. Thus, while the CDC and NCBI Philadelphia-1 genomes were closely related to each other, they were set apart from the remaining historical sequences and the ATCC Philadelphia-1 strain. Additionally, the Philadelphia-2, -3, and -4 isolates were separated by a nominal genetic distance; surprisingly, isolate E1-P fell within the Philadelphia-2/3/4 branch, but was recovered from California in 2013.

The kSNP-based, core-SNP tree was constructed with 11,356 unique nucleotide positions found in all sequences, but only 1 SNP was identified between isolates in both additional confirmed outbreaks (C3-O/E8-O and C11-O/E7-O), and 23 SNPs in the potential outbreak pair (C5-P/E10-P). With this method, from 3–13 SNP differences were found in pairwise comparisons of the Philadelphia-2, -3, -4, ATCC Philadelphia-1, and E1-P genomes. The NCBI Philadelphia-1 reference sequence and CDC Philadelphia-1 strain were highly similar to each other (3 SNPs), but they differed from the remaining Philadelphia historical isolates and the ATCC Philadelphia-1 strain by ≥541 core SNPs (data not shown). An average 1,277 core SNPs separated epidemiologically-unrelated *Legionella* isolates (S2 Fig), but this count ranged widely from 11 to 4,994 SNPs, with a large standard deviation (± 1,180 SNPs). Remarkably, fewer than 67 core SNPs were found in 66 of the 273 pairwise comparisons examined, and ≤20 core SNPs were identified in 5 separate comparisons encompassing 6 different unassociated strains.

### Whole-Genome Comparisons

Whole-genome alignments among isolates in the Philadelphia clade, including E1-P, demonstrated pairwise nucleotide identities of ≥98.35% (data not shown). Given the core-SNP-based phylogenetic similarity among these genomes, an unexpectedly large number of nucleotide polymorphisms were found (Table 2), and two observations were most striking: 1) A large nucleotide disparity (between 3,715 and 52,286 SNPs) separated the NCBI and CDC Philadelphia-1 sequences from all other genomes in the clade, and 2) the Philadelphia-2, -4 and ATCC Philadelphia-1 strains differed by ≤33 pairwise SNPs each.

**Table 2:** Pairwise whole-genome SNP comparisons of Philadelphia clade *L*. *pneumophila* isolates.

	# of Pairwise SNP Differences						
***L*. *pneumophila* strain**	**Philadelphia-1 (NCBI)**	**Philadelphia-1 (CDC)**	Philadelphia-2*	**Philadelphia-3**	Philadelphia-4*	Philadelphia-1 (ATCC)*	**E1-P**
**Philadelphia-1 (NCBI)**	X	12,032	14,393	14,577	14,396	14,386	16,538
**Philadelphia-1 (CDC)**	12,032	X	3,545	3,715	3,536	3,526	5,743
Philadelphia-2*	52,283	41,435	X	214	33	23	2,236
**Philadelphia-3**	14,577	3,715	38,104	X	205	195	2,410
Philadelphia-4*	52,286	41,426	33	38,095	X	14	2,231
Philadelphia-1 (ATCC)*	52,276	41,416	23	38,085	14	X	2,221
**E1-P**	16,538	5,743	40,126	2,410	40,121	40,111	

SNPs include insertions, deletions, and substitutions;

*, genomes contain pP36-Ph; Left/bottom diagonal half includes pP36-Ph where applicable; top/right diagonal half excludes pP36-Ph where applicable; Colors represent relative number of SNP differences, from maximum (red) to minimum (green)

**Fig 2:**
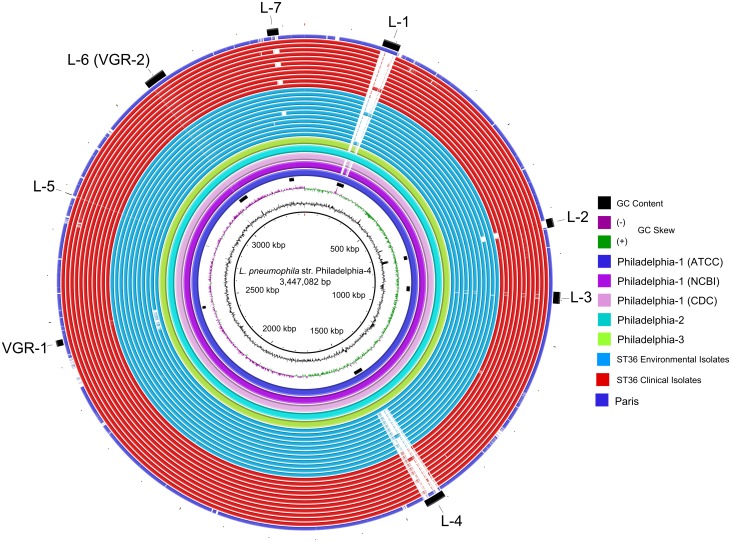
BRIG analysis plot comparing nucleotide content of all genomes analyzed in the current study. As defined in the legend, clinical isolates are shown in red while environmental isolates are blue. From the innermost ring: first black ring, *L*. *pneumophila* str. Phildadelphia-4 used as reference; second black ring, G+C content; third multicolored ring, GC skew; black bars, loci identified on the outer black bars and labeled; light blue ring 1 through 11 represent E1-P, E2-N, E3-N, E4-N, E6-N, E7-O, E8-O, E9-O, E10-P, and E11-U, respectively; red ring 1 through 11 represent C1-S, C2-S, C3-O, C4-S, C5-P, C6-S, C7-O, C8-S, C9-S, C10-S, and C11-O, respectively; outer purple ring *L*. *pneumophila* str. Paris; the outer black ring/bars highlight regions of interest, e.g., L-1 is locus 1, L-2 is locus 2, VGR-2 is variable genomic region 2, etc.

A BRIG analysis [31] revealed that approximately 97% of genome content within all 27 *Legionella* assemblies, plus the NCBI Philadelphia-1 and *L*. *pneumophila* str. Paris (NC_006368) reference sequences, was conserved in relation to strain Philadelphia-4 (Fig 2). Two large genomic regions, Locus-1 (L-1) and Locus-4 (L-4), and at least 5 additional, smaller regions (L-2, L-3 and L-5 –L-7) displayed variable degrees of conservation to the reference. L-1 was only present in the Philadelphia-2, -4, ATCC Philadelphia-1, and Paris strains, while L-4 was identified in all Philadelphia strains and isolate E1-P. Among the smaller loci were the *rtxA* toxin (L-2) and the sg1-specific 18-kb LPS biosynthesis region (L-3), both of which are associated with *Legionella* virulence [42, 43]. L-5 was identified as a conserved deletion of the ISSod13 transposase (lpg2421) in all additional ST36 genomes, with the exception of E1-P. The historical CDC Philadelphia strains encode 2 exact copies of this transposase which may be associated with intrachromosomal rearrangement. A second copy of ISSod13 (lpg1242) is located within the pLP45 virulence-associated genomic island [12]. The L-7 region contained genes for carbohydrate and amino acid metabolism, and cell envelope lipid synthesis; it also encoded various transcription regulatory elements, such as *rho*, and an ABC transporter.

**Fig 3:**
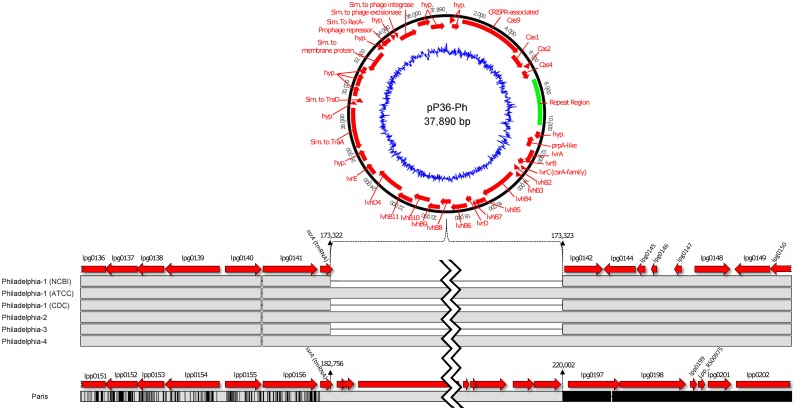
The pP36-Ph mobilizable genetic element. Nucleotide and structural comparison of the chromosomally integrated element within *L*. *pneumophila* strains Philadelphia and Paris, and the potential episomal form are shown. Gene prediction for pP36-Ph is based on the current strain Paris annotations found at NCBI. The inner blue circle of the episome represents G+C content. Horizontal, solid grey strips represent identical, conserved sequence, while thin vertical black lines or solid black regions represent nucleotide differences (SNPs) between genomes. Thin horizontal black lines between solid grey regions represent gaps or deletions in the sequence. The nucleotide boundaries where pP36-Ph is integrated in the chromosome are shown above the sequence (relative to strain Philadelphia-1 and Paris), along with neighboring genes. A double jagged line represents additional internal sequence not shown.

An in-depth analysis through the NCBI blastn suite (http://blast.ncbi.nlm.nih.gov/Blast.cgi) characterized the L-1 region as ~98% identical to a 36-kb “plasmid-like genomic island” that is reportedly mobilized from the *L*. *pneumophila* str. Paris chromosome to a circular, self-replicating form in a growth-phase-dependent manner [40, 44]. This episome, designated pP36 (here named pP36-Ph; Fig 3), has been found in all Paris-type sg1 strains examined, some non-Paris sg1 and non-sg1 isolates, but not in non-pneumophila species [45]. The average G+C content of the pP36-Ph element (44%) is higher than the strain Philadelphia chromosome (~38.3%), and it carries a potential virulence-associated *lvh/lvr* (*L**egionella* *v**ir* homologues) type IVA secretion/conjugation system composed of 16 conserved genes [46] similar to *Agrobacterium tumefaciens*, but distinct from the critical *dot/icm* type IVB system. pP36-Ph contains additional *traA*- and *traD*- conjugal transfer genes, a *csrA* homologue (*lvrC*), potential membrane proteins, putative prophage transcriptional repressors, and a *prpA-*like phage repressor. It also encodes a CRISPR system, which may act as a bacterial acquired immunity to foreign genetic material [47], with 4 Cas-like endonuclease genes near a ~3,000 bp repeat region that is longer than its strain Paris counterpart [39]; however, the full-sized repeat region may not be uncommon in the ST1/Paris-pulsotype [35]. pP36-Ph was identified in the Philadelphia-2, -4, and ATCC Philadelphia-1 strains (Fig 3), but not in other isolates sequenced here, and it accounted for most SNP differences in pairwise comparisons of these sequences (Table 2).

While pP36-Ph is described as “plasmid-like” due to its extrachromosomal replication, it also shares features with genomic islands, such as a dissimilar G+C content, and integrative conjugative elements (ICE), such as the ability to excise, circularize, and transfer to compatible recipients via the self-encoded conjugation machinery [48–50]. Several mobile genomic islands and ICEs have been described in *Legionella* that encode distinct but conserved type IVA conjugation systems, and at least two ICEs (apart from the pLP45 genomic island) have been described in strain Philadelphia-1, including pLP100 and ICE-βox (LpPI-1) [30, 40, 46, 51–54]. Similar to these mobile elements, pP36-Ph is integrated in a recombination hotspot between the tmRNA gene (*ssrA*) and 2 putative transposases (lpg0142 and lpg0144), and it is flanked by a 31-nt direct repeat that is the likely site of attachment (*att*). Notably, the strain Paris pP36 element and the strain Corby Trb2 mobile genomic island are also integrated at the same chromosomal locus. In our survey of the non-Philadelphia strains sequenced here, we also discovered a plasmid-like element of ~35.5-kb at the same genomic position in the E8-O and C3-O outbreak isolates (here named pO35TX; data not shown). p035TX has an elevated G+C content (40.7%) and *lvh/lvr* type IVA conjugation genes with high similarity to a virulence-associated locus in *L*. *pneumophila* str. AA100 [55].

Extrachromosomal mobilization of pP36-Ph was investigated when we observed a defined “spike” in both Pacbio and Illumina sequencing coverage of ~5-fold above average in the Philadelphia-2,-4, and ATCC Philadelphia-1 assemblies from coordinates 173,291 to 211,211, corresponding to the location of pP36-Ph. Individual Illumina sequencing reads that imperfectly aligned at the coverage spike boundaries were also found to partially align with sequence at the opposite ends of the coverage spike, both internal and external to the boundaries. This read-mapping arrangement was consistent with a circular DNA element, a properly re-ligated excision (or integration) site, and a chromosomally integrated form of pP36-Ph (data not shown), consistent with previous reports [40, 44]. The possibility of multiple, tandem pP36-Ph elements within the historical Philadelphia genomes was excluded after examination of corresponding optical maps (data not shown). A similar pattern of Illumina read partial alignment, suggesting episomal mobilization, was also observed for the pO35TX element in isolates E8-O and C3-O (data not shown).

The largest locus of interest identified by BRIG analysis, L-4, was conserved in isolate E1-P and all Philadelphia genomes, but not in the remaining ST36 assemblies; the region corresponded to the 45-kb plasmid-like element, pLP45, originally described by Chien, et.al. (2004) [12] extending from lpg1228 to lpg1271. This potential episome contains homologues for a phage repressor (*prp*), conjugal transfer proteins (*traA* and *traD*), and a putative recombinase, as well as an *lvh/lvr* locus that is ~92% identical to pP36-Ph. Unlike pP36-Ph, pLP45 encodes putative transposases, an SOS response transcriptional regulator, replication and repair proteins, a host restriction-modification system, protein symporter, and hypothetical ORFs apparently unique to *L*. *pneumophila*; pLP45, however, does not contain a CRISPR system. While the G+C content of the *lvh/lvr* region in this (44.2%) and other strains is elevated compared to the parent chromosome (~38.3%) [46], pLP45 as a whole is lower (37.5%). pLP45 was originally reported as episomal [12], however, we did not observe evidence for mobilization outside the chromosome or its complete excision as in *L*. *pneumophila* str. Lp01 [56].

**Fig 4:**
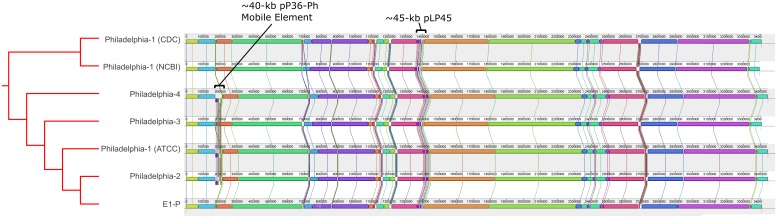
Mauve whole-genome alignment of *L*. *pneumophila* strains within the Philadelphia clade. ProgressiveMauve was used to compare the fully assembled sequences of the Philadelphia historical *Legionella* strains as well as isolate E1-P. The minimum weight for pairwise LCBs (locally collinear blocks), which share common colors across genomes, was set to 100, otherwise, the program was run using default parameters as described in the Methods. The general clade organization, as well as the identity and location of the ~40-kb pP36-Ph and the ~45-kb pLP45 elements are shown. The general, expanded Philadelphia clade organization from Fig 1 is shown, therefore the phylogenetic distances are not to scale.

A multiple whole-genome alignment using Mauve revealed largely syntenic locally collinear blocks (LCB) across most genomes (S3A Fig), consistent with previous studies [30, 39, 57]; notably, strain E1-P displayed strikingly similar genomic organization to all Philadelphia isolates (Fig 4). However, three substantial structural dissimilarities were found: 1) a ~40-kb LCB, similar in size and location to L-1, was identified in the first 210,000 bp of Philadelphia-2, -4, and ATCC strains, that was not found in the NCBI Philadelphia-1 reference, strains CDC Philadelphia-1, -3, or the remaining ST36 collection; 2) a ~45-kb insertion, equivalent to L-4, was identified in all Philadelphia strains, but was absent from the remaining ST36 isolates, except E1-P; and 3) a ~56-kb insertion was observed (at position ~2,668,000 relative to the NCBI Philadelphia-1 reference) in more than half of the additional ST36 genomes, but not in the Philadelphia clade isolates (S3B Fig).

The arrangement, genomic location, and nucleotide identity of the 56-kb region was highly conserved in 15 of the additional ST36 isolates. Approximately 16-kb of this insertion was similar to a “P-type” type IV conjugal DNA transfer system encoded within a *L*. *pneumophila* str. Lorraine genomic island (GI-Lo1) [30]. A comparably sized region was also found in *L*. *pneumophila* strains LPE509, 130b, Corby, Alcoy, and *L*. *longbeachae* NSW150 (data not shown). While the element was not conserved in any historical Philadelphia genome, the integration locus is structurally intact [30]. Non-*lvh/lvr* type IV secretion system components were also identified within all assembled plasmids, and in several ST36 genomes outside the 56-kb locus described above (data not shown). Isolates C1-S, E3-N, and E11-U contain large chromosomal insertions at a locus immediately upstream of the previously characterized ICE-βox element [52], all of which encode components of non-*lvh/lvr* type IV secretion/conjugation systems. A ~75-kb insertion at this position in isolate C1-S is highly similar to sequence within the *L*. *pneumophila* str. LPE509 chromosome, and insertions within the remaining isolates share similarity to genomic regions found in a number of different *L*. *pneumophila* and non-pneumophila strains (data not shown).

### Genetic Variation Among the Philadelphia Strains

**Fig 5:**
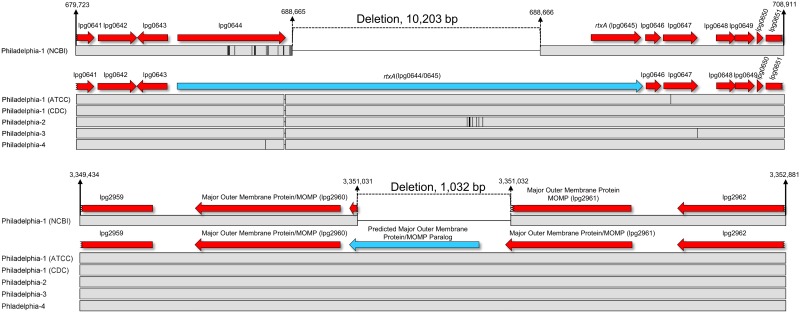
Selected genetic differences between the NCBI strain Philadelphia-1 reference sequence and all historical CDC Philadelphia isolates. Solid grey strips, and vertical and horizontal black lines represent conserved sequence, nucleotide SNPs, and sequence gaps, respectively, as described in Fig 3. Nucleotide boundaries for the potential *rtxA* and *mompS* deletions of ~10,203 and ~1,032 bp, respectively, are given above the sequence representations relative to the NCBI strain Philadelphia-1 reference sequence. The blue arrow in the top pane represents the full length *rtxA* gene found in strains Philadelphia-1, -2, -3, and -4. The blue arrow in the bottom pane represents a tandem *mompS* paralog not identified in the NCBI strain Philadelphia-1 reference sequence.

A detailed, pairwise whole-genome comparison uncovered substantial genetic differences, totaling ~12,032–52,286 bp, between the newly sequenced CDC Philadelphia strains and the NCBI Philadelphia-1 reference sequence (Table 2). As stated previously, the single largest genetic inconsistency within this collection is the ~38-kb pP36-Ph mobile element found only in strains Philadelphia-2, -4, and ATCC Philadelphia-1, however, a substantial number of pairwise SNPs still exist when pP36-Ph is removed from consideration. We identified 11,446 additional polymorphisms shared among the newly sequenced Philadelphia strains, including CDC Philadelphia-1, that were not found in the NCBI Philadelphia-1 reference (Table 3). These include a 158 nt indel upstream of the *iraAB* (lpg0746) iron assimilation operon, 2 nucleotide substitutions in a 16S rRNA gene (lpg2753), and SNPs in an LPS biosynthesis protein (lpg0748), a glycosyl transferase (lpg0775), and a glutamine amidotransferase (lpg2721) that result in non-conservative amino acid changes or frameshifts. We also confirmed that the historical CDC Philadelphia strains do not contain a previously described [56] single nucleotide insertion in the *rtxA* gene (lpg0644), as seen in the NCBI strain Philadelphia-1 reference, that creates a frameshift and early stop codon. RtxA is a human virulence-associated toxin [55] studied in select *Legionella* strains important for host cell entry [42, 58] as well as infection and trafficking within amoeba. Instead, the comparable region in the CDC Philadelphia-1, -2, -3, -4 and ATCC Philadelphia-1 genomes contains ~44 unreported SNPs and a 10,203 nt insertion that were potentially deleted from the NCBI Philadelphia-1 reference. A putative full length RtxA open reading frame of 6,420 amino acids results from the combination of lpg0644 and lpg0645, and encodes ~25 tandem repeats of ~536 nucleotides each, as opposed to the 6 repeats [59] in the NCBI reference (due to the frameshift) (Fig 5).

**Table 3:** Nucleotide polymorphisms shared by all CDC Philadelphia strains relative to the NCBI Philadelphia-1 reference sequence.

Nucleotide Polymorphism	No. of Polymorphisms (nt)	Nucleotide position	Nucleotide position in NCBI Philadelphia-1	Associated ORF's	Potential Translational Importance
Multiple SNPs	44	Ph-1: 685,979–688,613	685,979–688,614	*rtxA* (lpg0644)	Various indels and amino acid changes
		Ph-2: 723,869–726,503			
		Ph-3: 685,978–688,612			
		Ph-4: 723,869–726,503			
		ATCC Ph-1: 723,869–726,503			
Insertion	10,203	Ph-1: 688,664	688,665	*rtxA* (lpg0644-lpg0645)	Single, rtxA ORF of 6420 amino acids created from lpg0644 and lpg0655
		Ph-2: 726,554			
		Ph-3: 688,663			
		Ph-4: 726554			
		ATCC Ph-1: 726,554			
G → A	1	Ph-1: 826,712	816,510	Repeat region upstream of *iraAB* (lpg0746)	Unknown
		Ph-2: 864,602			
		Ph-3: 826,711			
		Ph-4: 864,602			
		ATCC Ph-1: 864,602			
Insertion	158	Ph-1: 826,741	816,539	Repeat region upstream of *iraAB* (lpg0746)	Unknown
		Ph-2: 864,631			
		Ph-3: 826,741			
		Ph-4: 826,741			
		ATCC Ph-1: 864,631			
T → G	1	Ph-1: 826,922	816,562	Repeat region upstream of *iraAB* (lpg0746)	Unknown
		Ph-2: 864,812			
		Ph-3: 826,921			
		Ph-4: 864,812			
		ATCC Ph-1: 864,812			
Multiple SNPs	2	Ph-1: 830,817	820,457	LPS biosynthesis protein, PseA-like (lpg0748)	V368R
		Ph-2: 868,707			
		Ph-3: 830,816			
		Ph-4: 868,707			
		ATCC Ph-1: 868,707			
Del G	1	Ph-1: 859,606	849,246	Glycosyl transferase (lpg0775)	Premature stop at codon 53, potential longer ORF created
		Ph-2: 897,496			
		Ph-3: 859,605			
		Ph-4: 897,496			
		ATCC Ph-1: 897,496			
N → T	1	Ph-1: 1,366,331	1,355,972	Hypothetical protein (lpg1228)	Synonymous
		Ph-2: 1,404,221			
		Ph-3: 1,366,329			
		Ph-4: 1,404,219			
		ATCC Ph-1: 1,404,221			
Del G	1	Ph-1: 3,083,758	3,073,980	Glutamine amidotransferase (lpg2721)	Frameshift creates premature stop at codon 34, potential upstream start creates longer ORF in with same lpg2721 frame
		Ph-2: 3,122,281			
		Ph-3: 3,084,386			
		Ph-4: 3,122,280			
		ATCC Ph-1: 3,122,280			
A → T	1	Ph-1: 3,111,785	3,102,008	16S rRNA (lpg2753)	Unknown
		Ph-2: 3,150,308			
		Ph-3: 3,112,411			
		Ph-4: 3,150,303			
		ATCC Ph-1: 3,150,307			
C → G	1	Ph-1: 3,111,912	3,102,135	16S rRNA (lpg2753)	Unknown
		Ph-2: 3,150,435			
		Ph-3: 3,112,538			
		Ph-4: 3,150,430			
		ATCC Ph-1: 3,150,434			
Insertion	1,032	Ph-1: 3,360,806	3,351,031	*mompS* (lpg2961)	Addition of tandem MOMPS ORF
		Ph-2: 3,499,329			
		Ph-3: 3,361,253			
		Ph-4: 3,399,330			
		ATCC Ph-1: 3,399,329			

Ph-1, CDC Philadelphia-1; Ph-2, Philadelphia-2; Ph-3, Philadelphia-3; Ph-4, Philadelphia-4; ATCC Ph-1, ATCC Philadelphia-1

The NCBI Philadelphia-1 reference sequence also contains a modest 1,032 bp deletion not found in any CDC historical genome, beginning at position ~3,351,031 that results in the apparent deletion of one of two tandem *mompS* paralogs (Fig 5). MompS is a *Legionella* cell surface virulence factor important for adherence [60] and entry [61] into phagocytes, and it is one of 7 genes included in the international sequence-based typing (SBT) scheme [62, 63]. When properly assembled, the 2 *mompS* paralogs are ~99% identical to each other, ~50% identical to a putative *momp* gene immediately upstream (lpg2960), and ~75% identical to a *momp* homologue found elsewhere in the chromosome (lpg1974). This *momp* gene complement and structural organization is conserved in all additional ST36 strains sequenced in the present study, as well as in all *Legionella* reference genomes examined (data not shown). The *mompS* sequence on which the SBT amplification and sequencing primers were designed (GenBank Accession AF078136) appears to be a hybrid containing unique sequence from the termini and intergenic regions surrounding these two tandem *mompS* paralogs. While not confirmed, it is possible that both the *rtxA* and *mompS* deletions in the NCBI Philadelphia-1 reference sequence are due to errors in sequencing and/or assembly, and not actual nucleotide variation. Apart from the shared polymorphisms listed above, the CDC Philadelphia-1 genome contains 586 additional nucleotide differences compared to the NCBI Philadelphia-1 reference, the majority of which are represented by a 582 nt in-frame deletion in a histidine kinase response regulator, lpg1912 (data not shown). While a subset of these nucleotide variants have been described previously [56], most are reported here for the first time.

**Fig 6:**
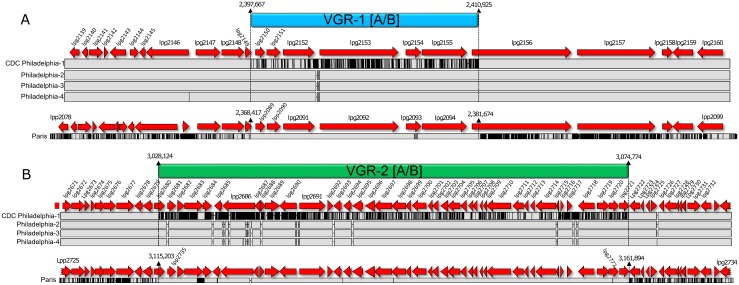
Variable genomic region 1 and 2 (VGR-1, and -2) within *L*. *pneumophila* Philadelphia strains and additional ST36 genomes. **(A)** VGR-1**A** is conserved in strain Philadelphia-1 (CDC) and in 19 of 22 additional ST36 strains, including strains C2-S, C3-O, C4-S, C5-P, C6-S, C7-O, C8-S, C9-S, C10-S, C11-O, E3-N, E4-N, E5-N, E6-N, E7-O, E8-O, E9-O, E10-P, E11-U, as well as *L*. *pneumophila* str. LPE509 and a sg12 strain (ATCC 43290), while VGR-1**B** is conserved in strains Philadelphia-2, -3, -4, E1-P, C1-S, and Paris. **(B)** VGR-2**A** is conserved in strain Philadelphia-1 (CDC) and in 21 of 22 additional ST36 strains, including C1-S, C2-S, C3-O, C4-S, C5-P, C6-S, C7-O, C8-S, C9-S, C10-S, C11-O, E2-N, E3-N, E4-N, E5-N, E6-N, E7-O, E8-O, E9-O, E10-P, and E11-U, while VGR-2**B** is conserved in strains Philadelphia-2, -3, -4, E1-P, and Paris. Solid grey strips, vertical, and horizontal black lines represent conserved sequence, SNPs, and sequence gaps, respectively, as in Fig 3. A Solid blue or green rectangle above the sequence delineates the boundaries of the VGR. Nucleotide boundaries and neighboring genes (in red) are relative to the genome immediately below these descriptions.

Consistent with our previous analyses, significant variation was also found *within* the CDC Philadelphia strain family. Most notable were 2,942 nt polymorphisms common to strains Philadelphia-2, -3, -4, and ATCC Philadelphia-1 that were not found in the CDC Philadelphia-1 sequence (Table 4). Four synonymous substitutions were documented, along with non-synonymous SNPs and indels located in genes for multi-drug efflux (lpg0662), a response-regulator (lpg1292), toxin secretion (lpg1515), a LysR family transcriptional regulator (lpg2288), a hypothetical protein (lpg0774), and within a 23S rRNA gene (lpg0571). By direct comparison to strain CDC Philadelphia-1, we observed that most nucleotide polymorphisms (besides pP36-Ph) shared among the Philadelphia-2, -3, -4, and ATCC Philadelphia-1 strains were localized in 2 highly variable genomic regions, VGR-1 and VGR-2 (L-6) (Fig 6). VGR-1 was ~13,300 bp in length, and encompassed ~541 SNPs within and between lpg2149 and lpg2156. This region contains 2 genes of unknown function (lpg2149 and lpg2150), with the former predicted as a Dot/Icm substrate [64], an aminoglycoside 6-adenylyltransferase (lpg2151) whose expression is upregulated in water, potentially for the development of the *Legionella* mature intracellular form [65], and a multidrug resistance ABC transporter ATP-binding protein (lpg2152). The remaining four genes in VGR-1 have been described as a “cluster”, encoding confirmed and predicted substrates of the Dot/Icm type IV secretion system, including *sdeC* (lpg2153), *laiE* (lpg2154), *sidJ* (lpg2155), and *sdeB* (lpg2156) [64, 66, 67]. Our identification of VGR-1 is consistent with a previous study that described a large hypervariable genomic region surrounding this locus, although it was not characterized further [68].

**Table 4:** Nucleotide polymorphisms shared by strains CDC Philadelphia-2, -3, -4, and ATCC Philadelphia-1 relative to the NCBI Philadelphia-1 reference sequence.

Nucleotide Polymorphism	No. of Polymorphisms (nt)	Nucleotide position	Nucleotide position in NCBI Philadelphia-1	Associated ORF's	Potential Translational Importance
C → T	1	Ph-2: 134,596	134,596	Glycine dehydrogenase subunit 1 (lpg0116)	Synonomous
		Ph-3: 134,597			
		Ph-4: 134,596			
		ATCC Ph-1: 134,596			
A → G	1	Ph-2: 648,327	610,437	23S rRNA (lpg0571)	Unknown
		Ph-3: 610,437			
		Ph-4: 648,327			
		ATCC Ph-1: 648,327			
C → T	1	Ph-2: 761,121	713,029	Major facilitator superfamily multidrug-efflux transporter (lpg0662)	T450I
		Ph-3: 723,230			
		Ph-4: 761,121			
		ATCC Ph-1: 761,121			
T → C	1	Ph-2: 896,048	847,798	Hypothetical protein (lpg0774)	Synonymous
		Ph-3: 858,157			
		Ph-4: 896,048			
		ATCC Ph-1: 896,048			
T → A	1	Ph-2: 1,468,719	1,420,470	DNA-binding response regulator (lpg1292)	H99L
		Ph-3: 1,430,827			
		Ph-4: 1,468,717			
		ATCC Ph-1: 1,468,719			
G → A	1	Ph-2: 1,636,663	1,588,414	Mg2+ and Co2+ transporter CorC (lpg1439)	Synonymous
		Ph-3: 1,598,772			
		Ph-4: 1,636,661			
		ATCC Ph-1: 1,636,663			
G → T	1	Ph-2: 1,726,062	1,677,813	Toxin secretion ATP-binding protein (lpg1515)	W387L
		Ph-3: 1,688,170			
		Ph-4: 1,726,060			
		ATCC Ph-1: 1,726,062			
T → G	1	Ph-2: 1,726,239	1,688,349	Toxin secretion ATP-binding protein (lpg1515)	`I446R
		Ph-3: 1,688,347			
		Ph-4: 1,726,237			
		ATCC Ph-1: 1,726,239			
Insertion	10	Ph-2: 1,726,519	1,678,270	Toxin secretion ATP-binding protein (lpg1515)	Framshift removes stop codon and creates fusion of lpg1515 and lpg1516
		Ph-3: 1,688,627			
		Ph-4: 1726,517			
		ATCC Ph-1: 1,726,519			
C → T	1	Ph-2: 2,370,109	2,321,850	Hypothetical protein (lpg2077)	G1102R
		Ph-3: 2,332,216			
		Ph-4: 2,370,106			
		ATCC Ph-1: 2,370,109			
VGR-1	541	Ph-2: 2,445,926–2,459,183	2,397,667–2,410,925	Region of high nucleotide variability (lpg2149-lpg2156)	Various Indels and substitutions
		2,408,033–2,421,290			
		Ph-4: 2,445,923–2,459,180			
		ATCC Ph-1: 2,445,926–2,459,183			
T → C	1	Ph-2: 2,592,341	2,544,083	Hypothetical protein (lpg2244)	Synonymous
		Ph-3: 2,554,448			
		Ph-4: 2,592,338			
		ATCC Ph-1: 2,592,341			
T → A	1	Ph-2: 2,638,436	2,590,178	LysR family transcriptional regulator (lpg2288)	F136I
		Ph-3: 2,600,543			
		Ph-4: 2,638,433			
		ATCC Ph-1: 2,638,436			
VGR-2	2,380	Ph-2: 3,076,382–3,123,074	3,028,124–3,074,774	Region of high nucleotide variability (lpg2680-lpg2721)	Various Indels and substitutions
		Ph-3: 3,038,486–3,085,179			
		Ph-4: 3,076,378–3,123,069			
		ATCC Ph-1: 3,076,382–3,123,073			

Ph-1, CDC Philadelphia-1; Ph-2, Philadelphia-2; Ph-3, Philadelphia-3; Ph-4, Philadelphia-4; ATCC Ph-1, ATCC Philadelphia-1

VGR-2 was ~47-kb in length, stretched from lpg2680 to lpg2721, and incorporated ~2,380 SNPs within 41 genes; a single tRNA (Val, lpg2715) located within this region did not contain nucleotide variants but was identical to the NCBI Philadelphia-1 reference. Similar to the smaller VGR-1, VGR-2 encompassed genes for confirmed and putative Dot/Icm substrates (hypothetical protein, lpg2692; *legD1*, lpg2694; *wipA*, lpg2718; and *legN*, lpg2720), but components of the Dot/Icm secretion apparatus itself (*dotA*, lpg2686; *icmX*, lpg2689; *icmW*, lpg2688; and *icmV*, lpg2687) were also found within [64, 68–73]. Additionally, VGR-2 housed genes for various metabolic and potential respiratory pathways (*amiB*, lpg2698; *petA*, lpg2705; *petB*, lpg2704; ubiquinol-cytochrome C reductase, lpg2703; sugar kinase, lpg2700; Iron-binding protein, lpg2708; and glutamine amidotransferase, lpg2721), replication and repair processes (*mutL*, lpg2697), stringent and starvation response (*sspA*, lpg2702 and *sspB*, lpg2701), ribosomal proteins (*rplT*, L20, lpg2712; *rplM*, L13, lpg2707; and *rpsI*, S9, lpg2706), other translation-associated genes (*thrS*, lpg2714; *pheS*, lpg2711; pheT, lpg2712; and *infC*, lpg2713), and at least 3 other potential virulence-associated genes (*murE3*, lpg2680; heptosyl transferase glycosyltransferase 9, lpg2695; and *infA*, lpg2709) [74–76].

Collectively, SNPs were accumulated within these VGRs at ~20-fold higher frequency compared to surrounding sequence, accounting for ~82% of the variation between strain CDC Philadelphia-1 and its sister strains, apart from pP36-Ph. Of the 1,663 single nucleotide variants within VGR-1 and -2 protein coding regions, ~69% were translationally silent or resulted in conservative amino acid substitutions, which is consistent with a previous study of one virulence-associated gene (*sidJ*) within VGR-1 [77]. Examination of VGR-1 flanking sequences revealed a remarkable concentration of potential and confirmed Dot/Icm secretion substrates both upstream (*legAU13*, lpg2144; *legA6*, lpg2131; *legK2*, lpg2137; *mavC*, lpg2147; hypothetical protein, lpg2148) and downstream (*sdeA/laiA*, lpg2157; hypothetical protein, lpg2160; *lem19*, lpg2166; and *legS2*, lpg2176) [64, 78–83]. VGR-2-downstream sequence also encodes potential Dot/Icm substrates (hypothetical proteins lpg2744 and lpg2745) [64], but more importantly, 3 additional components of the secretion apparatus are found immediately adjacent to this region (*dotB*, lpg2676; *dotC*, lpg2675; and *dotD*, lpg2674).

Interestingly, several genes bordering and within these regions encode type IV secretion substrates potentially acquired by interdomain horizontal gene transfer (HGT) [84], but if or how VGR-1 and -2 are actively mobilized is not known. Unlike traditional pathogenicity islands or ICEs, the G+C content of both VGRs is similar to the chromosome average (VGR-1 = 38.5% and VGR-2 = 38.9%), there are no apparent flanking repeat regions, and only a single transposase is located downstream of VGR-1 (lpg2173) near a tRNA (Lys, lpg2174). In addition, we did not observe evidence for mobilization of this region as we did for pP36-Ph or pO35TX. However, regions of elevated SNP density in the CDC Philadelphia strains are consistent with studies demonstrating frequent *Legionella* recombinational events [85], as well as selected genomic loci where most polymorphisms may accumulate in outbreak-associated isolates, ostensibly through HGT [86]. Thus, we queried our remaining ST36 genomic dataset and discovered that a Philadelphia-1 VGR-specific SNP pattern (VGR-1**A**) was conserved (>99.9%) in 19 additional genomes, as well as in *L*. *pneumophila* str. LPE509 and a sg12 strain (ATCC 43290) (Fig 6A); the Philadelphia-2/3/4 allele of this variable region (VGR-1**B**) was conserved in 2 ST36 genomes (E1-P and C1-S), plus strain Paris, and a single isolate (E2-N) lacked the homologous genomic region. Likewise, SNP distribution in the Philadelphia-1 VGR-2**A** region was preserved in 22 genomes, while VGR-2**B** from Philadelphia-2/3/4 was conserved in isolate E1-P plus strain Paris (Fig 6B). The C1-S genome, remarkably, was a hybrid of the Philadelphia-1 VGR-1**A**, and the Philadelphia-2/3/4 VGR-2**B**.

In addition to the genetic differences shared by all CDC Philadelphia isolates, or among the Philadelphia-2, -3, -4 and ATCC Philadelphia-1 sequences, a small number of unique polymorphisms were found in every Philadelphia strain (Table 5). Strain Philadelphia-3 contained the most unique polymorphisms (n = 13) while the ATCC Philadelphia-1 strain contained the fewest (n = 2). Unexpectedly, 25 out of 28 of these polymorphisms located within protein coding regions resulted in a frameshift, stop codon, or non-conservative amino acid substitution. It is also notable that CDC Philadelphia strains 1–4 all contain non-conserved, unique mutations within the phosphenolpyruvate protein-phosphotransferase gene *(ptsP*; lpg2871), and in 3 instances a frameshift and stop codon result. Mutations in *ptsP* do not affect growth on laboratory media but have been associated with growth defects within a human alveolar cell line and a guinea pig model of pneumonia [87].

**Table 5:** Nucleotide polymorphisms unique to the CDC Philadelphia strains relative to the NCBI Philadelphia-1 reference sequence.

Strain	Nucleotide Polymorphism	No. of Polymorphisms (nt)	Nucleotide position	Nucleotide position in NCBI Philadelphia-1	Associated ORF's	Potential Translational Importance
CDC Philadelphia-1	C → A	1	490,668	490,668	*icmG*(lpg0452)	Premature stop at codon 28
	C → T	1	894,870	884,511	Upstream of Na/H antiporter (lpg0806)	Unknown
	Deletion	582	2,143,988	2,133,629	Sensory box histidine kinase (lpg1912)	In-frame deletion
	Ins T	1	3,047,909	3,038,132	Upstream of *icmW* (lpg2688)	Unknown
	Del T	1	3,259,077	3,249,300	*ptsP* (lpg2871)	Premature stop at codon 340
Philadelphia-2	Insertion	37,890	173,321	173,321	Downstream of *ssrA* (tmRNA)	Chromosomal integration of pP36-Ph, shared with Philadelphia-4 and ATCC Philadelphia-1
	G → T	1	418,480	380,590	*fusA*(lpg0326)	G299C
	T → C	1	944,447	896,198	*clpA*(lpg0818)	F95S
	T → A	1	1,081,176	1,032,927	*tldD* (lpg0951)	L87M
	T → C	1	1,504,897	1,456,648	*cyaA*(lpg1322)	Y485C
	C →T	1	1,562,303	1,514,054	Hypothetical protein (lpg1368)	Synonymous
	G → A	1	2,134,556	2,086,297	tRNA-Leu (lpg1863)	Unknown
	Del T	1	3,297,382	3,249,082	*ptsP* (lpg2871)	Framshift creates premature stop at codon 375
Philadelphia-3	Ins T	1	12,618	12,618	hypothetical protein (lpg0008)	Framshift creates premature stop at codon 98
	G → A	1	142,453	142,452	Cytochrom C4 (lpg0124)	Premature stop at codon 116
	Del G	1	609,681	609,680	16S rRNA (lpg0569)	Unknown
	Del G	1	611,552	611,552	23S rRNA (lpg0571)	Unknown
	C → T	1	705,342	695,141	Hypothetical protein (lpg0647)	L441F
	T → C	1	746,429	736,228	Hypothetical protein (lpg0684)	F441L
	Del T	1	1,030,855	1,020,497	Hypothetical protein (lpg0941)	Premature stop at codon 749
	G → A	1	1,127,583	1,117,226	Chemiosmotic efflux system B protein A (lpg1020)	A876T
	Ins T	1	1,433,538	1,423,181	Hydroxyacylglutathione hydrolase GloB (lpg1295)	Frameshift changes protein sequence and adds 2 amino acids
	Del T	1	2,144,052	2,133,685	Sensory box histidine kinase (lpg1912)	Framshift causes premature stop at codon 38
	Del T	1	3,088,004	3,077,599	Hypothetical protein (lpg2725)	Frameshift creates premature stop at codon 204
	G → T	1	3,151,187	3,140,784	*nuoB2* (lpg2788)	Synonymous
	Deletion	181	3,258,530	3,248,127	*ptsP* (lpg2871)	Frameshift creates premature stop at codon 631
Philadelphia-4	G → A	1	7,181	7,181	Intergenic region between lpg004-lpg005	Unknown
	Insertion	37,890	173,321	173,321	Downstream of *ssrA* (tmRNA)	Chromosomal integration of pP36-Ph, shared with Philadelphia-2 and ATCC Philadelphia-1
	G → T	1	40,4828	36,6938	*secE* (lpg0316)	L55F
	G → A	1	420,902	383,012	*rpsJ/nusE* (lpg0328)	G34S
	T → C	1	2,163,269	2,115,012	Glutathione-regulated potassium efflux system (lpg1897)	L7P
	C → A	1	2,442,338	2,394,082	Downstream of *tutC* response regulator (lpg2146)	Unknown
	A → G	1	2,539,995	2,491,740	Downstream of hypothetical protein (lpg2207)	Unknown
	Insertion	2	3,125,898	3,077,599	Hypothetical protein (lpg2725)	Frameshift creates premature stop at codon 194
	A → G	1	3,197,722	3,149,427	*ftsH* (lpg2796)	I478T
	T → C	1	3,296,986	3,248,468	*ptsP* (lpg2871)	Q495R
ATCC Philadelphia-1	Insertion	37,890	173,321	173,321	Downstream of ssrA (tmRNA)	Chromosomal integration of pP36-Ph, shared with Philadelphia-2 and -4
	C → T	1	742,136	694,044	Hypothetical protein (lpg0647)	T75I
	C → A	1	3,042,468	2,994,210	*gacA* response regulator (lpg2646)	R74L

Ph-1, CDC Philadelphia-1; Ph-2, Philadelphia-2; Ph-3, Philadelphia-3; Ph-4, Philadelphia-4; ATCC Ph-1, ATCC Philadelphia-1

**Fig 7:**
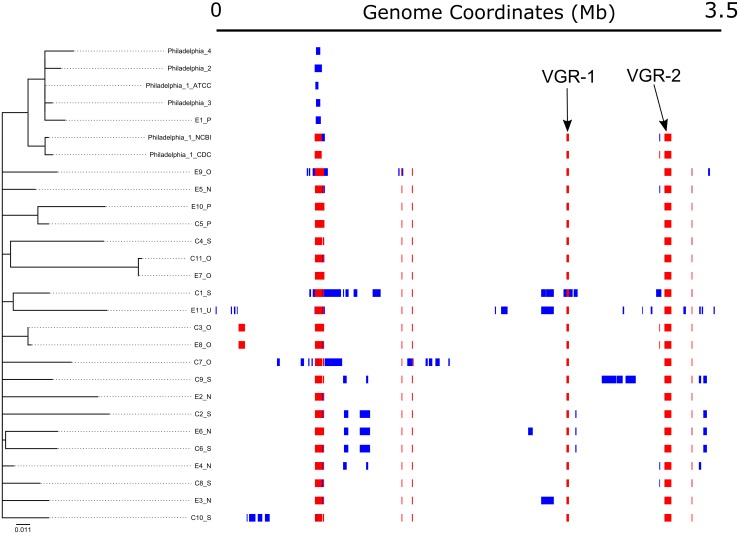
Gubbins-based recombinational analysis and phylogenetic tree reconstruction. Regions of elevated SNP density, representing potential horizontal gene transfer events, were identified by the Gubbins algorithm and software package, masked in the original multiple sequence alignment, and then core SNPs were identified with kSNP v3, as detailed in the Methods. The maximum-likelihood tree shown was constructed using RAxML v8 and 307 core, non-recombinant SNPs with 1000 bootstrappings. Red blocks represent regions of elevated SNP density conserved in multiple strains and blue blocks represent elevated SNP density only found in a single strain. *L*. *pneumophila* str. Philadelphia-4 was used as the reference/outgroup, and VGR-1 and -2 are labeled. Gubbins was run with default parameters. A Gubbins-generated tree with 480 non-recombining SNPs also exhibited similar topology.

Large-scale nucleotide variation introduced by high-frequency recombination may complicate interpretations of phylogenetic trees based on occasional single-base substitutions [86]. Therefore, we applied an algorithm developed by Croucher, *et*. *al* (2015) through the Gubbins software package, combined with a modified kSNP pipeline, to detect potential recombinational events in our genomic dataset [36]. The increased SNP density observed within VGR-1 and VGR-2 were confirmed, with high probability (negative log likelihood = 1226.2 and 5845.3, respectively), to be indicative of HGT. Importantly, a maximum-likelihood phylogeny constructed with only the non-recombinant core SNPs reflected the Philadelphia clade topology seen in the previous core-SNP and core-gene trees (Fig 7); the confirmed outbreak-associated isolates (C3-O/E8-O and C11-O/E7-O) also clustered, despite overall lower resolution. We identified only a small number of non-recombinant core SNPs (n = 2–17) among the strain Philadelphia genomes with this method, and the confirmed outbreak pairs differed by 1 SNP each. Importantly, the non-VGR polymorphisms common to strains Philadelphia -2, -3, -4, and ATCC Philadelphia-1 (n = 12) were not found to be associated with HGT.

## Discussion

Both the scale of the 1976 Philadelphia LD outbreak and subsequent CDC public health response were, to that point in time, unprecedented. Historical records, congressional testimony, and related correspondence attest to a major mobilization of human and laboratory resources that led to the recovery of 4 clinical *L*. *pneumophila* isolates. These strains formed the basis of the first diagnostic tests for Legionnaires’ disease [5, 15, 88–90], and the first isolate, Philadelphia-1, assumed a prominent role in microbiologic, epidemiologic, diagnostic, and environmental research as the prototype for this novel genus and species. The present study was a detailed analysis of these outbreak-associated isolates by whole-genome sequencing (WGS).

We discovered that the NCBI Phildaelphia-1 reference sequence and strain CDC Philadelphia-1 are derived from a close common ancestor; the Philadelphia-2, -3, -4, and ATCC Philadelphia-1 isolates are highly similar, and together, all Philadelphia strains comprise a shared genetic lineage. However, the CDC Philadelphia-1 and -3 strains lack a plasmid-like element found in the remaining isolates, and CDC Philadelphia-2, -3, -4 and ATCC strains contain >2,900 SNPs, compared to CDC Philadelphia-1, concentrated in two highly variable genomic regions (VGR-1 and -2). Both core-gene and SNP-based phylogenetic analyses placed all Philadelphia isolates within the same clade, but the CDC and NCBI Philadelphia-1 strains were situated on their own distinct branch, separated from the remaining Philadelphia strains by an evolutionary distance that is larger than expected for isogenic outbreak strains. As determined here, the *L*. *pneumophila* str. Philadelphia family represents a clonal group where traditional background mutation, gene deletion, episomal loss/acquisition, and HGT have led to varying degrees of genetic divergence; thus, isolate CDC Philadelphia-1 (and the NCBI Philadelphia-1 sequence strain) should not be considered identical to the remaining outbreak-associated strains Philadelphia-2, -3, or -4. We also confirmed the identity of ATCC strain Philadelphia-1 that has previously been called into question [56]; this isolate did originate from the 1976 outbreak but is most similar to, and likely derived from either strain Philadelphia-2 or -4 isolated at the CDC.

The functional consequences of polymorphisms identified in this study remain unexplored, but several observations based on protein coding predictions are notable. As described above, most substitutions located within VGR-1 and -2 were silent or gave rise to conserved amino acid changes suggesting that proteins such as the virulence-associated type IV secretion component DotA, and several ribosomal proteins, have important cellular functions. Three polymorphisms shared among the non-Philadelphia-1 strains cluster within a single gene annotated as “toxin secretion ATP-binding protein” (lpg1515). A 10 nt insertion in this gene shifts the reading frame to create a fusion with the downstream “ABC transporter ATP-binding/permease protein”, lpg1516, resulting in a potential 736 amino acid protein (or 718 amino acids if using an alternative start). This substantially larger protein is similar in size and pairwise identity to comparable annotated gene products in other sequenced legionellae (e.g., strain Paris and ATCC 43290). Additionally, a single SNP unique to the CDC Philadelphia-1 strain appears to create a premature stop at codon 28 in the *icmG*/*dotF* gene. A recent study of DotF suggested it was not an essential component of the Dot/Icm type IV secretion system, but serves an accessory function during intracellular growth within *Acanthamoeba castellanii* [91].

*Legionella* was not recovered from hotel A or the surrounding area despite a wide-ranging environmental investigation following the 1976 epidemic, thus it was not possible to confirm the precise source(s) of infection. Case epidemiology and our newly resolved phylogeny may, however, provide valuable insight: Philadelphia strains -2, -3, and -4 were recovered from American Legion conventioneers or relatives who entered hotel A on more than one occasion, but strain CDC Philadelphia-1 was isolated from a person with Broad Street pneumonia who never entered the convention hotel. By genetic comparison, the Broad Street pneumonia case was infected by a similar, yet distinct strain of *L*. *pneumophila* sg1 compared to the bacteria infecting the 3 hotel-associated victims. Collectively, these data suggest that multiple potential exposure sources could have existed in this urban environment, as observed in recent outbreaks and reports [92, 93]. Alternatively, several genetically related *Legionella* strains may have disseminated from a single contaminated source, a premise also supported by past outbreak investigations [94, 95]. We also observed that the Philadelphia isolates differed by 2–17 core, non-recombining SNPs, while both additional confirmed outbreak pairs diverged by a single core SNP. Based on a recent estimate of the core mutation rate for an outbreak-associated ST578 population [86] (~0.39 substitutions per genome per year), the Philadelphia strains may have diverged from a common ancestor many years before the 1976 epidemic, and could have persisted in or around hotel A for a significant period of time. Such long-term *Legionella* persistence has been widely reported [95–100]. Indeed, a smaller scale pneumonia outbreak with LD-compatible features occurred in the summer of 1974, 2 years prior to the 1976 Legion gathering, at the same Philadelphia hotel [101]. Four out of eleven potential LD cases from that epidemic demonstrated high *Legionella* antibody titers in 1977, suggestive of recent infection by a Philadelphia-type strain.

Plausibly, genetic variation found in strain CDC Philadelphia-1, but not its sister strains, could have resulted from a stressful initial propagation. This hypothesis is inconsistent, however, with several bodies of data, including an experimental evolution study [102], and a comparison of 2 domesticated Philadelphia-1 lineages that found only limited nucleotide changes compared to the published NCBI Philadelphia-1 reference [56]. Short-term passage experiments in our laboratory also confirmed that few polymorphisms arise in this time frame, and the pP36-Ph mobile element is stably maintained (data not shown). Direct genetic evidence includes the conservation and distribution of VGR-1 and VGR-2 sub-types (“A” and “B”) among the current ST36 strains, confirming that most variation between strain CDC Philadelpia-1 and its sister genomes, apart from pP36-Ph, is due to HGT rather than single nucleotide substitution [85]. Additional VGR sub-types beyond those identified here certainly exist, as several alleles of *dotA* and *sidJ* have been previously reported within VGR-2 and -1, respectively [77, 103]. Lastly, we documented 12 non-HGT-associated polymorphisms shared only among strains Philadelphia-2, -3, and -4 (Table 4), which argues against the hypothesis that variation within the Philadelphia clade arose after clinical isolation, but strongly supports a prior split in this lineage.

Few publications describe the use of whole-genome sequence analysis for *Legionella* phylogeny construction either retrospectively or in real-time during outbreaks [104–110]; thus, there is no established SNP threshold for outbreak-associated isolates. SNP-based approaches have typically found ≤20 SNPs between epidemiologically-linked strains, but upwards of ~200 SNPs have been reported [104, 106, 109]. In the present study, 20 or fewer core SNPs were identified in comparisons of 6 unrelated strains (S2 Fig). And unexpectedly, isolate E1-P clustered within the Philadelphia-2/3/4 branch with ≤13 core SNPs (Fig 1B) while sharing remarkably similar genomic organization (Fig 4). Yet, a full genome sequence comparison demonstrated ~2,265 pairwise SNPs in the E1-P *rtxA* region, and lack of the pP36-Ph episome (data not shown). Most critical, however, was the absence of epidemiological data linking strain E1-P with any other presently sequenced isolate. These results suggest that a “Philadelphia-type” genome may be widely distributed or common in parts of the United States and other countries, in agreement with previous findings [111]. Higher resolution genetic tools (e.g., long-read sequencing and full chromosome assembly) might be required in these select cases to clarify genotypic relationships initially established by SNP-based analyses. Importantly, as the E1-P isolate illustrates, phylogenetic interpretations in outbreak investigations that employ WGS should be made within an epidemiological context.

The present study is the first to employ both long and short-read sequencing for the assembly of complete *Legionella* genomes of the same sequence type as a genetic basis for comparison of potential outbreak isolates. These methods resolved complex genomic loci, such as extended variable repeats in the CRISPR and *rtxA* loci, and tandem paralogous *mompS* genes, and provided direct comparisons of entire chromosomes and genomic synteny. Our analysis revealed that all presently sequenced strains contain the necessary Dot/Icm type IVB secretion components (data not shown), but only strain E1-P, the confirmed outbreak pair C11-O/E7-O, and the historical Philadelphia isolates, house *lvh/lvr-*based type IVA systems (Fig 1A). And as detailed here, Philadelphia strains -2 and -4 harbor 2 *lvh/lvr* loci on the independent pP36-Ph and pLP45 episomes. While initially thought to be dispensable [46], this secretion apparatus can conditionally rescue defects in host cell entry, phagosome acidification, and replication caused by *dot*/*icm* mutation [112, 113]; and some *lvh/lvr* secretion components are important for cell invasion at low temperatures [114]. Thus, the presence of one or more *lvh/lvr* loci may suggest unique pathogenic potential by Philadelphia-type strains in the same fashion as ICE-βox confers resistance to oxidative stress and β-lactam antibiotics [52].

With faster, more accessible sequencing technologies, *Legionella* typing and outbreak-focused phylogenetic methods are being developed to utilize whole-genome data sets [104–109, 115]. Based on these novel approaches, this project defined the genetic and epidemiological relationships among a subset of historically significant *Legionella* strains while helping to establish a high-resolution *Legionella* outbreak pipeline. We discovered that the widely-studied *L*. *pneumophila* str. Philadelphia-1 originated from a case of Broad Street pneumonia in 1976, and although part of the same genetic lineage, it is distinct from the remaining Philadelphia outbreak-associated strains. The closed, whole-genome sequences published within also contribute to the body of data and public health resources available for the development and testing of novel *Legionella* diagnostics and outbreak databases.

## Supporting Information

S1 Fig Core-gene-based phylogeny of all *L*. *pneumophila* isolates examined in the present study. A maximum-likelihood tree was constructed using RAxML v8 and 2,699 core genes identified by orthologous ORF clustering, with 1000 bootstrappings, as described in the Methods. The NCBI strain Philadelphia-1 reference sequence is also included. The Philadelphia historical clade is colored red, while blue shaded boxes highlight the confirmed (-O) outbreak isolate pairs, and a green shaded box highlights the potential (-P) outbreak isolate pair. Units of branch length (“Tree Scale”) are in nucleotide substitutions per site.
(PDF)

S2 Fig SNP comparisons among epidemiologically unassociated *L*. *pneumophila* sg1 (ST36) isolates sequenced in the present study. A total of 273 pairwise, core-SNP-based comparisons were extracted from the initial kSNP core analysis and categorized into 20-SNP ranges. All epidemiologically linked or confirmed comparisons were removed, and from among all historical Philadelphia isolates only strains CDC Philadelphia-1 and -2 were included in the analysis. The blue line represents a skewed distribution of the mean for the included pairwise comparisons (mean = 1,277 core SNPs; median = 1,023 core SNPs; SD ± 1,180 core SNPs).
(PDF)

S3 Fig Mauve whole-genome alignment of all *L*. *pneumophila* isolates examined in the present study. **(A)** ProgressiveMauve was used to compare complete, assembled genomes, including plasmids, as well as NCBI strains Philadelphia-1 and Paris reference sequences. Parameters for Mauve were the same as for Fig 4. **(B)** A ~56-kb region from the larger alignment in “**A”** was found in 15 of 22 ST36 strains but not in any historical Philadelphia isolate.
(PDF)

S1 Table Genome characteristics and metadata of *L*. *pneumophila* sg1 (ST36) strains sequenced in the present study. (PDF)

## References

[1] Fraser DW, Tsai TR, Orenstein W, Parkin WE, Beecham HJ, Sharrar RG, et al Legionnaires' disease: description of an epidemic of pneumonia. N Engl J Med. 1977;297(22):1189–97. 10.1056/NEJM197712012972201 335244

[2] Hyatt D, Chen G-L, LoCascio PF, Land ML, Larimer FW, Hauser LJ. Prodigal: prokaryotic gene recognition and translation initiation site identification. BMC Bioinformatics. 2010;11(1):1–11. 10.1186/1471-2105-11-119 20211023 PMC2848648

[3] Pendergrast M. Inside the outbreaks: the elite medical detectives of the epidemic intelligence service. Boston: Houghton Mifflin Harcourt; 2010 xiv, 418 p.

[4] Legionnaires' disease, 1977: hearing before the Subcommittee on Health and Scientific Research of the Committee on Human Resources, United States Senate, Ninety-fifth Congress, first session… November 9, 1977. In: United, editor. Washington: U.S. Govt. Print. Off.; 1978. p. iii-161.

[5] McDade JE, Shepard CC, Fraser DW, Tsai TR, Redus MA, Dowdle WR. Legionnaires' disease: isolation of a bacterium and demonstration of its role in other respiratory disease. N Engl J Med. 1977;297(22):1197–203. 10.1056/NEJM197712012972202 335245

[6] Euzeby JP. List of Bacterial Names with Standing in Nomenclature: a folder available on the Internet. Int J Syst Bacteriol. 1997;47(2):590–2. 10.1099/00207713-47-2-590 9103655

[7] Coredes LG, Fraser DW, Skaliy P, Perlino CA, Elsea WR, Mallison GF, et al Legionnaires' Disease outbreak at an Atlanta, Georgia, Country Club: Evidence for spread from an evaporative condenser. Am J Epidemiol. 1980;111(4):425–31. 7377185 10.1093/oxfordjournals.aje.a112917

[8] Dondero TJ Jr, Rendtorff RC, Mallison GF, Weeks RM, Levy JS, Wong EW, et al An outbreak of Legionnaires' disease associated with a contaminated air-conditioning cooling tower. N Engl J Med. 1980;302(7):365–70. 10.1056/NEJM198002143020703 7351928

[9] Fraser DW. Legionellosis: evidence of airborne transmission. Ann N Y Acad Sci. 1980;353:61–6. 10.1111/j.1749-6632.1980.tb18906.x 6939401

[10] Morris GK, Patton CM, Feeley JC, Johnson SE, Gorman G, Martin WT, et al Isolation of the Legionnaires' Disease Bacterium from Environmental Samples. Ann Intern Med. 1979;90(4):664–6. 10.7326/0003-4819-90-4-664 373549

[11] Politi BD, Fraser DW, Mallison GF, Mohatt JV, Morris GK, Patton CM, et al A Major Focus of Legionnaires' Disease in Bloomington, Indiana. Ann Intern Med. 1979;90(4):587–91. 10.7326/0003-4819-90-4-587 434640

[12] Chien M, Morozova I, Shi S, Sheng H, Chen J, Gomez SM, et al The genomic sequence of the accidental pathogen *Legionella pneumophila*. Science. 2004;305(5692):1966–8. 10.1126/science.1099776 15448271

[13] Selander RK, McKinney RM, Whittam TS, Bibb WF, Brenner DJ, Nolte FS, et al Genetic structure of populations of *Legionella pneumophila*. J Bacteriol. 1985;163(3):1021–37. 4030689 10.1128/jb.163.3.1021-1037.1985 PMC219234

[14] Moss CW, Weaver RE, Dees SB, Cherry WB. Cellular fatty acid composition of isolates from Legionnaires disease. J Clin Microbiol. 1977;6(2):140–3. 893658 10.1128/jcm.6.2.140-143.1977 PMC274721

[15] Cherry WB, Pittman B, Harris PP, Hebert GA, Thomason BM, Thacker L, et al Detection of Legionnaires disease bacteria by direct immunofluorescent staining. J Clin Microbiol. 1978;8(3):329–38. 359594 10.1128/jcm.8.3.329-338.1978 PMC275241

[16] McKinney RM, Thacker L, Harris PP, Lewallen KR, Hebert GA, Edelstein PH, et al Four Serogroups of Legionnaires' Disease Bacteria Defined by Direct Immunofluorescence. Ann Intern Med. 1979;90(4):621–4. 10.7326/0003-4819-90-4-621 86313

[17] Brenner DJ, Steigerwalt AG, Weaver RE, McDade JE, Feeley JC, Mandel M. Classification of the Legionnaires' disease bacterium: An interim report. Curr Microbiol. 1978;1(2):71–5. 10.1007/BF02605418

[18] Feeley JC, Gibson RJ, Gorman GW, Langford NC, Rasheed JK, Mackel DC, et al Charcoal-yeast extract agar: primary isolation medium for *Legionella pneumophila*. J Clin Microbiol. 1979;10(4):437–41. 393713 10.1128/jcm.10.4.437-441.1979 PMC273193

[19] Pasculle AW, Feeley JC, Gibson RJ, Cordes LG, Myerowitz RL, Patton CM, et al Pittsburgh pneumonia agent: direct isolation from human lung tissue. J Infect Dis. 1980;141(6):727–32. 10.1093/infdis/141.6.727 7391615

[20] Edelstein PH. Improved semiselective medium for isolation of *Legionella pneumophila* from contaminated clinical and environmental specimens. J Clin Microbiol. 1981;14(3):298–303. 7287886 10.1128/jcm.14.3.298-303.1981 PMC271958

[21] Chin CS, Alexander DH, Marks P, Klammer AA, Drake J, Heiner C, et al Nonhybrid, finished microbial genome assemblies from long-read SMRT sequencing data. Nat Methods. 2013;10(6):563–9. 10.1038/nmeth.2474 23644548

[22] Ribeiro FJ, Przybylski D, Yin S, Sharpe T, Gnerre S, Abouelleil A, et al Finished bacterial genomes from shotgun sequence data. Genome Res. 2012;22(11):2270–7. 10.1101/gr.141515.112 22829535 PMC3483556

[23] Krumsiek J, Arnold R, Rattei T. Gepard: a rapid and sensitive tool for creating dotplots on genome scale. Bioinformatics. 2007;23(8):1026–8. 10.1093/bioinformatics/btm039 17309896

[24] Langmead B, Trapnell C, Pop M, Salzberg SL. Ultrafast and memory-efficient alignment of short DNA sequences to the human genome. Genome Biol. 2009;10(3):R25 10.1186/gb-2009-10-3-r25 19261174 PMC2690996

[25] Li H, Handsaker B, Wysoker A, Fennell T, Ruan J, Homer N, et al The Sequence Alignment/Map format and SAMtools. Bioinformatics. 2009;25(16):2078–9. 10.1093/bioinformatics/btp352 19505943 PMC2723002

[26] Li H. A statistical framework for SNP calling, mutation discovery, association mapping and population genetical parameter estimation from sequencing data. Bioinformatics. 2011;27(21):2987–93. 10.1093/bioinformatics/btr509 21903627 PMC3198575

[27] Danecek P, Auton A, Abecasis G, Albers CA, Banks E, DePristo MA, et al The variant call format and VCFtools. Bioinformatics. 2011;27(15):2156–8. 10.1093/bioinformatics/btr330 21653522 PMC3137218

[28] Darling AE, Mau B, Perna NT. progressiveMauve: multiple genome alignment with gene gain, loss and rearrangement. PLoS One. 2010;5(6):e11147 10.1371/journal.pone.0011147 20593022 PMC2892488

[29] Katoh K, Standley DM. MAFFT Multiple Sequence Alignment Software Version 7: Improvements in Performance and Usability. Mol Biol Evol. 2013;30(4):772–80. 10.1093/molbev/mst010 23329690 PMC3603318

[30] Gomez-Valero L, Rusniok C, Jarraud S, Vacherie B, Rouy Z, Barbe V, et al Extensive recombination events and horizontal gene transfer shaped the *Legionella pneumophila* genomes. BMC Genomics. 2011;12(1):1–24. 10.1186/1471-2164-12-536 22044686 PMC3218107

[31] Alikhan NF, Petty NK, Ben Zakour NL, Beatson SA. BLAST Ring Image Generator (BRIG): simple prokaryote genome comparisons. BMC Genomics. 2011;12:402 10.1186/1471-2164-12-402 21824423 PMC3163573

[32] Morrison SS, Williams T, Cain A, Froelich B, Taylor C, Baker-Austin C, et al Pyrosequencing-based comparative genome analysis of Vibrio vulnificus environmental isolates. PLoS One. 2012;7(5):e37553 10.1371/journal.pone.0037553 22662170 PMC3360785

[33] Sievers F, Wilm A, Dineen D, Gibson TJ, Karplus K, Li W, et al Fast, scalable generation of high-quality protein multiple sequence alignments using Clustal Omega. Mol Syst Biol. 2011;7:539 10.1038/msb.2011.75 21988835 PMC3261699

[34] Stamatakis A. RAxML version 8: a tool for phylogenetic analysis and post-analysis of large phylogenies. Bioinformatics. 2014;30(9):1312–3. 10.1093/bioinformatics/btu033 24451623 PMC3998144

[35] Ginevra C, Jacotin N, Diancourt L, Guigon G, Arquilliere R, Meugnier H, et al *Legionella pneumophila* ST1/Paris-Pulsotype subtyping by spoligotyping. J Clin Microbiol. 2011 10.1128/JCM.06180-11 22205819 PMC3295150

[36] Croucher NJ, Page AJ, Connor TR, Delaney AJ, Keane JA, Bentley SD, et al Rapid phylogenetic analysis of large samples of recombinant bacterial whole genome sequences using Gubbins. Nucleic Acids Res. 2015;43(3):e15 10.1093/nar/gku1196 25414349 PMC4330336

[37] Letunic I, Bork P. Interactive Tree Of Life (iTOL): an online tool for phylogenetic tree display and annotation. Bioinformatics. 2007;23(1):127–8. 10.1093/bioinformatics/btl529 17050570

[38] Lennette EH, Schmidt NJ, American Public Health Association. Subcommittee on Diagnostic Procedures for Viral and Rickettsial Infections., American Public Health Association. Diagnostic procedures for viral and rickettsial infections. 4th ed. New York,: American Public Health Association; 1969 xx, 978 p.

[39] D'Auria G, Jimenez-Hernandez N, Peris-Bondia F, Moya A, Latorre A. *Legionella pneumophila* pangenome reveals strain-specific virulence factors. BMC Genomics. 2010;11:181 10.1186/1471-2164-11-181 20236513 PMC2859405

[40] Cazalet C, Rusniok C, Bruggemann H, Zidane N, Magnier A, Ma L, et al Evidence in the *Legionella pneumophila* genome for exploitation of host cell functions and high genome plasticity. Nat Genet. 2004;36(11):1165–73. 10.1038/ng1447 15467720

[41] Gardner SN, Slezak T, Hall BG. kSNP3.0: SNP detection and phylogenetic analysis of genomes without genome alignment or reference genome. Bioinformatics. 2015;31(17):2877–8. 10.1093/bioinformatics/btv271 25913206

[42] Cirillo SLG, Lum J, Cirillo JD. Identification of novel loci involved in entry by *Legionella pneumophila*. Microbiology. 2000;146(6):1345–59. 10.1099/00221287-146-6-1345 10846213

[43] Petzold M, Thürmer A, Menzel S, Mouton JW, Heuner K, Lück C. A structural comparison of lipopolysaccharide biosynthesis loci of *Legionella pneumophila* serogroup 1 strains. BMC Microbiol. 2013;13(1):1–11. 10.1186/1471-2180-13-198 24069939 PMC3766260

[44] Doleans-Jordheim A, Akermi M, Ginevra C, Cazalet C, Kay E, Schneider D, et al Growth-phase-dependent mobility of the lvh-encoding region in *Legionella pneumophila* strain Paris. Microbiology. 2006;152(Pt 12):3561–8. 10.1099/mic.0.29227-0 17159208

[45] Luck C, Brzuszkiewicz E, Rydzewski K, Koshkolda T, Sarnow K, Essig A, et al Subtyping of the *Legionella pneumophila* "Ulm" outbreak strain using the CRISPR-Cas system. Int J Med Microbiol. 2015;305(8):828–37. 10.1016/j.ijmm.2015.08.001 26294350

[46] Segal G, Russo JJ, Shuman HA. Relationships between a new type IV secretion system and the icm/dot virulence system of *Legionella pneumophila*. Mol Microbiol. 1999;34(4):799–809. 10.1046/j.1365-2958.1999.01642.x 10564519

[47] Barrangou R, Fremaux C, Deveau H, Richards M, Boyaval P, Moineau S, et al CRISPR Provides Acquired Resistance Against Viruses in Prokaryotes. Science. 2007;315(5819):1709–12. 10.1126/science.1138140 17379808

[48] Wozniak RAF, Waldor MK. Integrative and conjugative elements: mosaic mobile genetic elements enabling dynamic lateral gene flow. Nat Rev Micro. 2010;8(8):552–63. 10.1038/nrmicro2382 20601965

[49] Johnson CM, Grossman AD. Integrative and Conjugative Elements (ICEs): What They Do and How They Work. Annu Rev Genet. 2015;49(1):577–601. 10.1146/annurev-genet-112414-055018 26473380 PMC5180612

[50] Boyd EF, Almagro-Moreno S, Parent MA. Genomic islands are dynamic, ancient integrative elements in bacterial evolution. Trends Microbiol. 2009;17(2):47–53. 10.1016/j.tim.2008.11.003 19162481

[51] Glöckner G, Albert-Weissenberger C, Weinmann E, Jacobi S, Schunder E, Steinert M, et al Identification and characterization of a new conjugation/type IVA secretion system (trb/tra) of *Legionella pneumophila* Corby localized on two mobile genomic islands. Int J Med Microbiol. 2008;298(5–6):411–28. 10.1016/j.ijmm.2007.07.012 17888731

[52] Flynn KJ, Swanson MS. Integrative Conjugative Element ICE-βox Confers Oxidative Stress Resistance to *Legionella pneumophila* In Vitro and in Macrophages. mBio. 2014;5(3):e01091–14. 10.1128/mBio.01091-14 24781744 PMC4010831

[53] Trigui H, Dudyk P, Sum J, Shuman HA, Faucher SP. Analysis of the transcriptome of Legionella pneumophila hfq mutant reveals a new mobile genetic element. Microbiology. 2013;159(8):1649–60. 10.1099/mic.0.067983-0 23728622 PMC5972305

[54] Brassinga AKC, Hiltz MF, Sisson GR, Morash MG, Hill N, Garduno E, et al A 65-Kilobase Pathogenicity Island Is Unique to Philadelphia-1 Strains of *Legionella pneumophila*. J Bacteriol. 2003;185(15):4630–7. 10.1128/JB.185.15.4630-4637.2003 12867476 PMC165780

[55] Samrakandi MM, Cirillo SLG, Ridenour DA, Bermudez LE, Cirillo JD. Genetic and Phenotypic Differences between *Legionella pneumophila* Strains. J Clin Microbiol. 2002;40(4):1352–62. 10.1128/JCM.40.4.1352-1362.2002 11923356 PMC140379

[56] Rao C, Benhabib H, Ensminger AW. Phylogenetic reconstruction of the *Legionella pneumophila* Philadelphia-1 laboratory strains through comparative genomics. PLoS One. 2013;8(5):e64129 10.1371/journal.pone.0064129 23717549 PMC3661481

[57] Gomez-Valero L, Rusniok C, Buchrieser C. *Legionella pneumophila*: population genetics, phylogeny and genomics. Infect Genet Evol. 2009;9(5):727–39. 10.1016/j.meegid.2009.05.004 19450709

[58] Cirillo SL, Bermudez LE, El-Etr SH, Duhamel GE, Cirillo JD. *Legionella pneumophila* entry gene *rtxA* is involved in virulence. Infect Immun. 2001;69(1):508–17. 10.1128/IAI.69.1.508-517.2001 11119544 PMC97910

[59] D'Auria G, Jiménez N, Peris-Bondia F, Pelaz C, Latorre A, Moya A. Virulence factor *rtx* in *Legionella pneumophila*, evidence suggesting it is a modular multifunctional protein. BMC Genomics. 2008;9(1):1–7. 10.1186/1471-2164-9-14 18194518 PMC2257941

[60] Krinos C, High AS, Rodgers FG. Role of the 25 kDa major outer membrane protein of *Legionella pneumophila* in attachment to U-937 cells and its potential as a virulence factor for chick embryos. J Appl Microbiol. 1999;86(2):237–44. 10.1046/j.1365-2672.1999.00667.x 10063623

[61] Bellinger-Kawahara C, Horwitz MA. Complement component C3 fixes selectively to the major outer membrane protein (MOMP) of *Legionella pneumophila* and mediates phagocytosis of liposome-MOMP complexes by human monocytes. The Journal of Experimental Medicine. 1990;172(4):1201–10. 10.1084/jem.172.4.1201 2212949 PMC2188623

[62] Gaia V, Fry NK, Afshar B, Lück PC, Meugnier H, Etienne J, et al Consensus Sequence-Based Scheme for Epidemiological Typing of Clinical and Environmental Isolates of *Legionella pneumophila*. J Clin Microbiol. 2005;43(5):2047–52. 10.1128/JCM.43.5.2047-2052.2005 15872220 PMC1153775

[63] Gaia V, Fry NK, Harrison TG, Peduzzi R. Sequence-Based Typing of *Legionella pneumophila* Serogroup 1 Offers the Potential for True Portability in Legionellosis Outbreak Investigation. J Clin Microbiol. 2003;41(7):2932–9. 10.1128/JCM.41.7.2932-2939.2003 12843023 PMC165343

[64] Gomez-Valero L, Rusniok C, Cazalet C, Buchrieser C. Comparative and Functional Genomics of *Legionella* Identified Eukaryotic Like Proteins as Key Players in Host–Pathogen Interactions. Front Microbiol. 2011;2:208 10.3389/fmicb.2011.00208 22059087 PMC3203374

[65] Li L, Mendis N, Trigui H, Faucher SP. Transcriptomic changes of Legionella pneumophila in water. BMC Genomics. 2015;16:637 10.1186/s12864-015-1869-6 26306795 PMC4549902

[66] Liu Y, Luo Z-Q. The Legionella pneumophila Effector SidJ Is Required for Efficient Recruitment of Endoplasmic Reticulum Proteins to the Bacterial Phagosome. Infect Immun. 2007;75(2):592–603. 10.1128/IAI.01278-06 17101649 PMC1828518

[67] Luo ZQ, Isberg RR. Multiple substrates of the Legionella pneumophila Dot/Icm system identified by interbacterial protein transfer. Proc Natl Acad Sci U S A. 2004;101(3):841–6. 10.1073/pnas.0304916101 14715899 PMC321768

[68] Zusman T, Degtyar E, Segal G. Identification of a Hypervariable Region Containing New Legionella pneumophila Icm/Dot Translocated Substrates by Using the Conserved icmQ Regulatory Signature. Infect Immun. 2008;76(10):4581–91. 10.1128/IAI.00337-08 18694969 PMC2546816

[69] Coers J, Kagan JC, Matthews M, Nagai H, Zuckman DM, Roy CR. Identification of Icm protein complexes that play distinct roles in the biogenesis of an organelle permissive for Legionella pneumophila intracellular growth. Mol Microbiol. 2000;38(4):719–36. 10.1046/j.1365-2958.2000.02176.x 11115108

[70] Ninio S, Zuckman-Cholon DM, Cambronne ED, Roy CR. The Legionella IcmS-IcmW protein complex is important for Dot/Icm-mediated protein translocation. Mol Microbiol. 2005;55(3):912–26. 10.1111/j.1365-2958.2004.04435.x 15661013

[71] Roy CR, Isberg RR. Topology of Legionella pneumophila DotA: an inner membrane protein required for replication in macrophages. Infect Immun. 1997;65(2):571–8. 9009315 10.1128/iai.65.2.571-578.1997 PMC176098

[72] Marra A, Blander SJ, Horwitz MA, Shuman HA. Identification of a Legionella pneumophila locus required for intracellular multiplication in human macrophages. Proceedings of the National Academy of Sciences. 1992;89(20):9607–11. 10.1073/pnas.89.20.9607 1409673 PMC50181

[73] Vogel JP, Andrews HL, Wong SK, Isberg RR. Conjugative Transfer by the Virulence System of Legionella pneumophila. Science. 1998;279(5352):873–6. 10.1126/science.279.5352.873 9452389

[74] Shevchuk O, Jager J, Steinert M. Virulence properties of the legionella pneumophila cell envelope. Front Microbiol. 2011;2:74 10.3389/fmicb.2011.00074 21747794 PMC3129009

[75] Morash MG, Brassinga AK, Warthan M, Gourabathini P, Garduno RA, Goodman SD, et al Reciprocal expression of integration host factor and HU in the developmental cycle and infectivity of Legionella pneumophila. Appl Environ Microbiol. 2009;75(7):1826–37. 10.1128/AEM.02756-08 19201975 PMC2663186

[76] Aurass P, Pless B, Rydzewski K, Holland G, Bannert N, Flieger A. bdhA-patD operon as a virulence determinant, revealed by a novel large-scale approach for identification of Legionella pneumophila mutants defective for amoeba infection. Appl Environ Microbiol. 2009;75(13):4506–15. 10.1128/AEM.00187-09 19411431 PMC2704810

[77] Costa J, Teixeira PG, d'Avó AF, Júnior CS, Veríssimo A. Intragenic Recombination Has a Critical Role on the Evolution of *Legionella pneumophila* Virulence-Related Effector *sidJ*. PLoS One. 2014;9(10):e109840 10.1371/journal.pone.0109840 25299187 PMC4192588

[78] Brüggemann H, Cazalet C, Buchrieser C. Adaptation of Legionella pneumophila to the host environment: role of protein secretion, effectors and eukaryotic-like proteins. Curr Opin Microbiol. 2006;9(1):86–94. 10.1016/j.mib.2005.12.009 16406773

[79] Huang L, Boyd D, Amyot WM, Hempstead AD, Luo Z-Q, O'Connor TJ, et al The E Block motif is associated with Legionella pneumophila translocated substrates. Cell Microbiol. 2011;13(2):227–45. 10.1111/j.1462-5822.2010.01531.x 20880356 PMC3096851

[80] de Felipe KS, Glover RT, Charpentier X, Anderson OR, Reyes M, Pericone CD, et al *Legionella* Eukaryotic-Like Type IV Substrates Interfere with Organelle Trafficking. PLoS Pathog. 2008;4(8):e1000117 10.1371/journal.ppat.1000117 18670632 PMC2475511

[81] Bardill JP, Miller JL, Vogel JP. IcmS-dependent translocation of SdeA into macrophages by the Legionella pneumophila type IV secretion system. Mol Microbiol. 2005;56(1):90–103. 10.1111/j.1365-2958.2005.04539.x 15773981

[82] Lurie-Weinberger MN, Gomez-Valero L, Merault N, Glöckner G, Buchrieser C, Gophna U. The origins of eukaryotic-like proteins in Legionella pneumophila. Int J Med Microbiol. 2010;300(7):470–81. 10.1016/j.ijmm.2010.04.016 20537944

[83] Degtyar E, Zusman T, Ehrlich M, Segal G. A Legionella effector acquired from protozoa is involved in sphingolipids metabolism and is targeted to the host cell mitochondria. Cell Microbiol. 2009;11(8):1219–35. 10.1111/j.1462-5822.2009.01328.x 19438520

[84] de Felipe KS, Pampou S, Jovanovic OS, Pericone CD, Ye SF, Kalachikov S, et al Evidence for Acquisition of *Legionella* Type IV Secretion Substrates via Interdomain Horizontal Gene Transfer. J Bacteriol. 2005;187(22):7716–26. 10.1128/JB.187.22.7716-7726.2005 16267296 PMC1280299

[85] Coscollá M, Comas I, González-Candelas F. Quantifying Nonvertical Inheritance in the Evolution of *Legionella pneumophila*. Mol Biol Evol. 2011;28(2):985–1001. 10.1093/molbev/msq278 20961962

[86] Sanchez-Buso L, Comas I, Jorques G, Gonzalez-Candelas F. Recombination drives genome evolution in outbreak-related *Legionella pneumophila* isolates. Nat Genet. 2014;46(11):1205–11. 10.1038/ng.3114 25282102

[87] Higa F, Edelstein PH. Potential Virulence Role of the Legionella pneumophila ptsP Ortholog. Infect Immun. 2001;69(8):4782–9. 10.1128/IAI.69.8.4782-4789.2001 11447151 PMC98565

[88] Farshy CE, Klein GC, Feeley JC. Detection of antibodies to legionnaires disease organism by microagglutination and micro-enzyme-linked immunosorbent assay tests. J Clin Microbiol. 1978;7(4):327–31. 357440 10.1128/jcm.7.4.327-331.1978 PMC274955

[89] Berdal BP, Farshy CE, Feeley JC. Detection of *Legionella pneumonophila* antigen in urine by enzyme-linked immunospecific assay. J Clin Microbiol. 1979;9(5):575–8. 383744 10.1128/jcm.9.5.575-578.1979 PMC275350

[90] Tilton RC. Legionnaires' Disease Antigen Detected by Enzyme-Linked Immunosorbent Assay. Ann Intern Med. 1979;90(4):697–8. 10.7326/0003-4819-90-4-697 434657

[91] Sutherland MC, Binder KA, Cualing PY, Vogel JP. Reassessing the Role of DotF in the *Legionella pneumophila* Type IV Secretion System. PLoS One. 2013;8(6):e65529 10.1371/journal.pone.0065529 23762385 PMC3676331

[92] O'Loughlin RE, Kightlinger L, Werpy MC, Brown E, Stevens V, Hepper C, et al Restaurant outbreak of Legionnaires' disease associated with a decorative fountain: an environmental and case-control study. BMC Infect Dis. 2007;7:93 10.1186/1471-2334-7-93 17688692 PMC1976126

[93] Bentham HR. Routine Sampling and the Control of *Legionella* spp. inCooling Tower Water Systems. Curr Microbiol.41(4):271–5. 10.1007/s002840010133 10977895

[94] Kioski C, Cage G, Johnson B, Rosales C, England B. Sustained transmission of nosocomial Legionnaires disease—Arizona and Ohio. MMWR. 1997;46(19):416–21. 9162842

[95] Kool JL, Fiore AE, Kioski CM, Brown EW, Benson RF, Pruckler JM, et al More than 10 years of unrecognized nosocomial transmission of legionnaires' disease among transplant patients. Infect Control Hosp Epidemiol. 1998;19(12):898–904. 10.2307/30142014 9872525

[96] Lepine LA, Jernigan DB, Butler JC, Pruckler JM, Benson RF, Kim G, et al A recurrent outbreak of nosocomial legionnaires' disease detected by urinary antigen testing: evidence for long-term colonization of a hospital plumbing system. Infect Control Hosp Epidemiol. 1998;19(12):905–10. 10.1017/S0195941700092092 9872526

[97] Rangel-Frausto MS, Rhomberg P, Hollis RJ, Pfaller MA, Wenzel RP, Helms CM, et al Persistence of *Legionella pneumophila* in a hospital's water system: a 13-year survey. Infect Control Hosp Epidemiol. 1999;20(12):793–7. 10.1086/501586 10614601

[98] Perola O, Kauppinen J, Kusnetsov J, KÄRkkÄInen U-M, LÜCk PC, Katila M-L. Persistent *Legionella pneumophila* colonization of a hospital water supply: efficacy of control methods and a molecular epidemiological analysis. APMIS. 2005;113(1):45–53. 10.1111/j.1600-0463.2005.apm1130107.x 15676014

[99] Cooper IR, White J, Mahenthiralingam E, Hanlon GW. Long-term persistence of a single *Legionella pneumophila* strain possessing the mip gene in a municipal shower despite repeated cycles of chlorination. J Hosp Infect. 2008;70(2):154–9. 10.1016/j.jhin.2008.06.015 18723253

[100] Silk BJ, Moore MR, Bergtholdt M, Gorwitz RJ, Kozak NA, Tha MM, et al Eight years of Legionnaires' disease transmission in travellers to a condominium complex in Las Vegas, Nevada. Epidemiol Infect. 2012;140(11):1993–2002. 10.1017/S0950268811002779 22214820

[101] Terranova W, Cohen M, Fraser D. 1974 Outbreak of Legionnaires' Disease Diagnosed in 1977: Clinical and Epidemiological Features. Lancet. 1978;312(8081):122–4. 10.1016/S0140-6736(78)91507-6 78324

[102] Ensminger AW, Yassin Y, Miron A, Isberg RR. Experimental evolution of *Legionella pneumophila* in mouse macrophages leads to strains with altered determinants of environmental survival. PLoS Pathog. 2012;8(5):e1002731 10.1371/journal.ppat.1002731 22693450 PMC3364954

[103] Bumbaugh CA, McGraw AE, Page LK, Selander KR, Whittam ST. Sequence Polymorphism of *dotA* and mip Alleles Mediating Invasion and Intracellular Replication of *Legionella pneumophila*. Curr Microbiol.44(5):314–22. 10.1007/s00284-001-0024-6 11927981

[104] Bartley PB, Ben Zakour NL, Stanton-Cook M, Muguli R, Prado L, Garnys V, et al Hospital-wide Eradication of a Nosocomial *Legionella pneumophila* Serogroup 1 Outbreak. Clin Infect Dis. 2015 10.1093/cid/civ870 26462745

[105] Bosch T, Euser SM, Landman F, Bruin JP, EP IJ, den Boer JW, et al Whole-Genome Mapping as a Novel High-Resolution Typing Tool for *Legionella pneumophila*. J Clin Microbiol. 2015;53(10):3234–8. 10.1128/JCM.01369-15 26202110 PMC4572561

[106] Graham RM, Doyle CJ, Jennison AV. Real-time investigation of a *Legionella pneumophila* outbreak using whole genome sequencing. Epidemiol Infect. 2014:epub ahead of print. 10.1017/S0950268814000375 24576553 PMC9151283

[107] Levesque S, Plante PL, Mendis N, Cantin P, Marchand G, Charest H, et al Genomic characterization of a large outbreak of *Legionella pneumophila* serogroup 1 strains in Quebec City, 2012. PLoS One. 2014;9(8):e103852 10.1371/journal.pone.0103852 25105285 PMC4126679

[108] Moran-Gilad J, Prior K, Yakunin E, Harrison TG, Underwood A, Lazarovitch T, et al Design and application of a core genome multilocus sequence typing scheme for investigation of Legionnaires' disease incidents. Euro Surveill. 2015;20(28). 10.2807/1560-7917.ES2015.20.28.21186 26212142

[109] Reuter S, Harrison TG, Koser CU, Ellington MJ, Smith GP, Parkhill J, et al A pilot study of rapid whole-genome sequencing for the investigation of a *Legionella* outbreak. BMJ Open. 2013;3(1):e002175 10.1136/bmjopen-2012-002175 23306006 PMC3553392

[110] Bartley PB, Ben Zakour NL, Stanton-Cook M, Muguli R, Prado L, Garnys V, et al Hospital-wide Eradication of a Nosocomial *Legionella pneumophila* Serogroup 1 Outbreak. Clin Infect Dis. 2016;62(3):273–9. 10.1093/cid/civ870 26462745

[111] Cazalet C, Jarraud S, Ghavi-Helm Y, Kunst F, Glaser P, Etienne J, et al Multigenome analysis identifies a worldwide distributed epidemic *Legionella pneumophila* clone that emerged within a highly diverse species. Genome Res. 2008;18 10.1101/gr.7229808 18256241 PMC2259107

[112] Bandyopadhyay P, Liu S, Gabbai CB, Venitelli Z, Steinman HM. Environmental Mimics and the Lvh Type IVA Secretion System Contribute to Virulence-Related Phenotypes of *Legionella pneumophila*. Infect Immun. 2007;75(2):723–35. 10.1128/IAI.00956-06 17101653 PMC1828514

[113] Bandyopadhyay P, Lang EAS, Rasaputra KS, Steinman HM. Implication of the VirD4 Coupling Protein of the Lvh Type 4 Secretion System in Virulence Phenotypes of *Legionella pneumophila*. J Bacteriol. 2013;195(15):3468–75. 10.1128/JB.00430-13 23729650 PMC3719543

[114] Ridenour DA, Cirillo SLG, Feng S, Samrakandi MM, Cirillo JD. Identification of a Gene That Affects the Efficiency of Host Cell Infection by *Legionella pneumophila* in a Temperature-Dependent Fashion. Infect Immun. 2003;71(11):6256–63. 10.1128/IAI.71.11.6256-6263.2003 14573644 PMC219575

[115] Gilmour MW, Bernard K, Tracz DM, Olson AB, Corbett CR, Burdz T, et al Molecular typing of a *Legionella pneumophila* outbreak in Ontario, Canada. J Med Microbiol. 2007;56(Pt 3):336–41. 10.1099/jmm.0.46738-0 17314363 PMC2884934

