# Supplementary figures and images for: Genomic Analysis Reveals Novel Diversity among the 1976 Philadelphia Legionnaires’ Disease Outbreak Isolates and Additional ST36 Strains
Source: PLoS One. 2016 Sep 29;11(9):e0164074. doi: 10.1371/journal.pone.0164074 (PMC5042515; doi:10.1371/journal.pone.0164074)

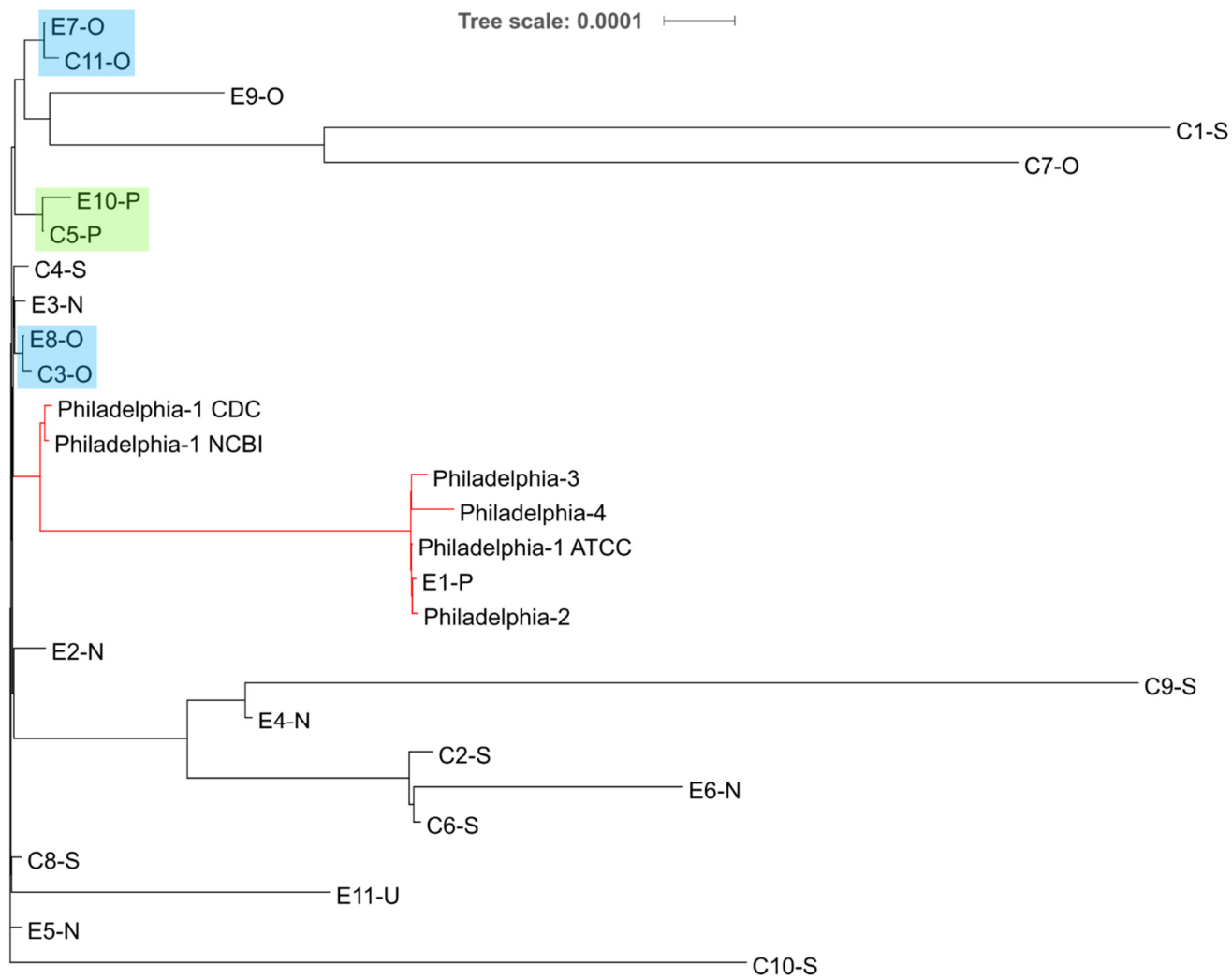

**S1 Fig.** Core gene-based phylogeny of all *L. pneumophila* isolates examined in the present study.

Supplement: S1 Fig — A maximum-likelihood tree was constructed using RAxML v8 and 2,699 core genes identified by orthologous ORF clustering, with 1000 bootstrappings, as described in the Methods. The NCBI strain Philadelphia-1 reference sequence is also included. The Philadelphia historical clade is colored red, while blue shaded boxes highlight the confirmed (-O) outbreak isolate pairs, and a green shaded box highlights the potential (-P) outbreak isolate pair. Units of branch length (“Tree Scale”) are in nucleotide substitutions per site. (PDF) [file pone.0164074.s001.pdf]

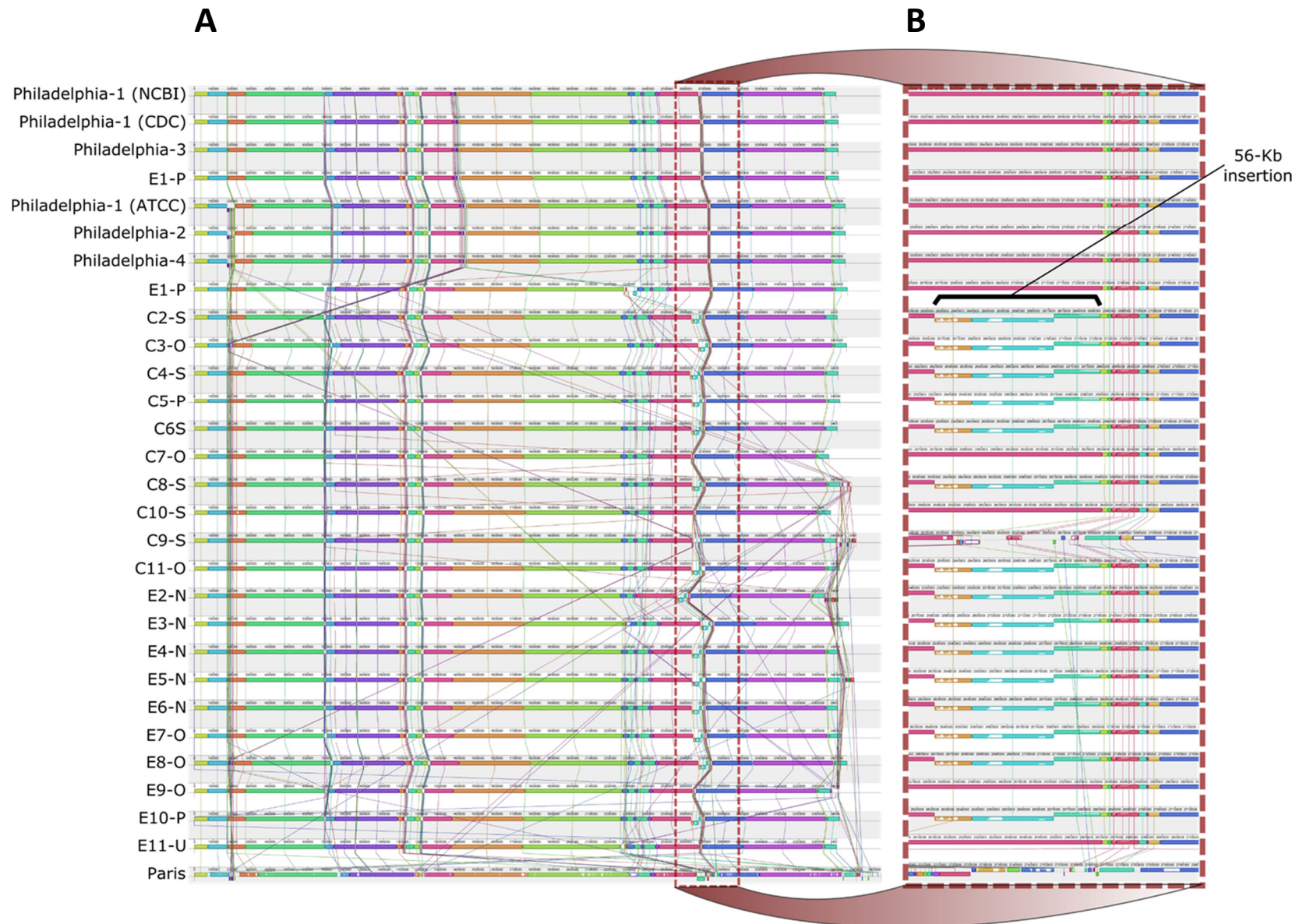

**S3 Fig. Mauve whole-genome alignment of all *L. pneumophila* isolates examined in the present study.**

Supplement: S3 Fig — (A) ProgressiveMauve was used to compare complete, assembled genomes, including plasmids, as well as NCBI strains Philadelphia-1 and Paris reference sequences. Parameters for Mauve were the same as for Fig 4. (B) A ~56-kb region from the larger alignment in “A” was found in 15 of 22 ST36 strains but not in any historical Philadelphia isolate. (PDF) [file pone.0164074.s003.pdf]
