# Supplementary material for: Genomic Analysis Reveals Novel Diversity among the 1976 Philadelphia Legionnaires’ Disease Outbreak Isolates and Additional ST36 Strains
Source: PLoS One. 2016 Sep 29;11(9):e0164074. doi: 10.1371/journal.pone.0164074 (PMC5042515; doi:10.1371/journal.pone.0164074)
Supplement: S2 Fig — A total of 273 pairwise, core-SNP-based comparisons were extracted from the initial kSNP core analysis and categorized into 20-SNP ranges. All epidemiologically linked or confirmed comparisons were removed, and from among all historical Philadelphia isolates only strains CDC Philadelphia-1 and -2 were included in the analysis. The blue line represents a skewed distribution of the mean for the included pairwise comparisons (mean = 1,277 core SNPs; median = 1,023 core SNPs; SD ± 1,180 core SNPs). (PDF) [file pone.0164074.s002.pdf]

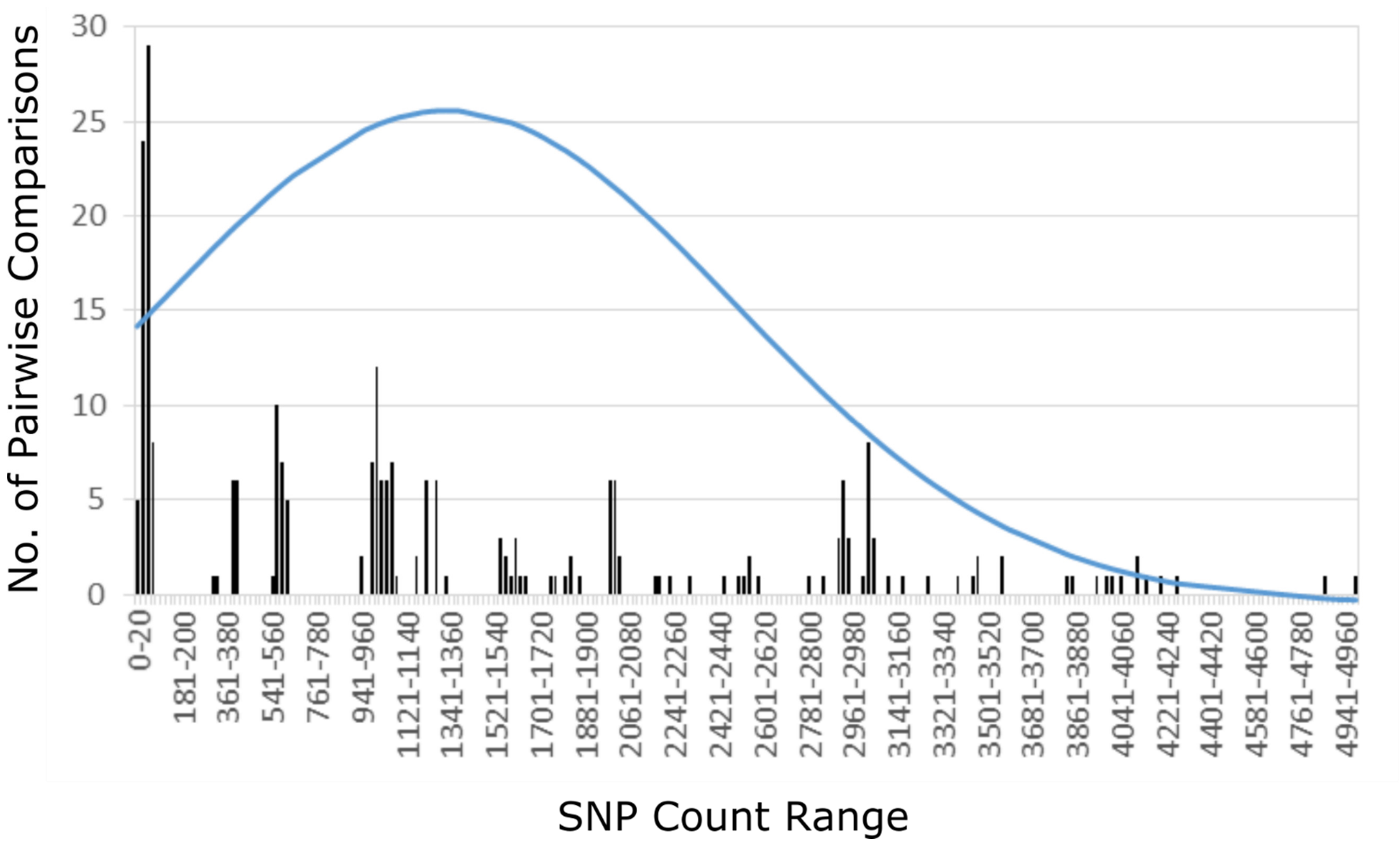

S2 Fig. SNP comparisons among epidemiologically unassociated *L. pneumophila* sg1 (ST36) isolates sequenced in the present study.
