# Supplementary material for: Genomic Analysis Reveals Novel Diversity among the 1976 Philadelphia Legionnaires’ Disease Outbreak Isolates and Additional ST36 Strains
Source: PLoS One. 2016 Sep 29;11(9):e0164074. doi: 10.1371/journal.pone.0164074 (PMC5042515; doi:10.1371/journal.pone.0164074)
Supplement: S1 Table — (PDF) [file pone.0164074.s004.pdf]

S1 Table. Genome characteristics and metadata of *L. pneumophila* sg1 (ST36) strains sequenced in the present study.

Strain or Isolate Designation	State of Origin	Source of Isolate	Association	Date Originally Isolated or Received	Assembled Contigs	G+C Content (%)	Main Choromosome Size (bp)	No. of Putative Protein Coding Genes	Plasmids, No. (size in bp/G+C %)	NCBI Accession (PacBio chr./plas./SRA)	References/Notes
Philadelphia-1 CDC	Pennsylvania	Autopsy lung tissue	Outbreak	1/11/1977	1	38.33	3,408,562	3024	0	CP015928/ SRR3613186	McDade, et. al. (1977); Fraser, et. al. (1977)
Philadelphia-2	Pennsylvania	Autopsy lung tissue	Outbreak	1/11/1977	1	38.35	3,447,085	3062	1* (37,889/40.02)	CP015929/ SRR3613187	McDade, et. al. (1977); Fraser, et. al. (1977)
Philadelphia-3	Pennsylvania	Autopsy lung tissue	Outbreak	1/16/1977	1	38.33	3,409,008	3024	0	CP015930/ SRR3613188	McDade, et. al. (1977); Fraser, et. al. (1977)
Philadelphia-4	Pennsylvania	Autopsy lung tissue	Outbreak	1/16/1977	1	38.35	3,447,082	3062	1* (37,889/40.02)	CP015931/ SRR3613189	McDade, et. al. (1977); Fraser, et. al. (1977)
Philadelphia-1 ATCC (33152)	Pennsylvania	Autopsy lung tissue	Outbreak	1/11/1977	1	38.35	3,447,084	3059	1* (37,889/40.02)	CP015927/ SRR3613190	McDade, et. al. (1977); Fraser, et. al. (1977)
C1-S	North Carolina	Clinical isolate, sputum	Sporadic	1/26/2009	1	38.38	3,457,332	3070	0	CP015932/ SRR3613171	Present study
C2-S	Illinois	Clinical isolate, TTA	Sporadic	11/6/2007	1	38.32	3,404,161	3004	0	CP015933/ SRR3613172	Present study
C3-O	Texas	Clinical isolate	Outbreak	6/7/2006	1	38.32	3,453,407	3064	1* /35,481/40.7	CP015934/ SRR3613183	Present study; Additional outbreak pair 2; Plasmid named here pO35TX
C4-S	Georgia	Clinical isolate, BAL	Sporadic	3/20/2000	1	38.26	3,409,361	3021	0	CP015935/ SRR3613191	Present study

C5-P	Ohio	Clinical isolate, unknown	Potential Outbreak	2/24/1998	1	38.31	3,419,718	3021	0	CP015936/ SRR3613192	Present study; Potential outbreak pair
C6-S	New Jersey	Clinical isolate, sputum	Sporadic	1/22/1996	1	38.32	3,404,196	3006	0	CP015937/ SRR3613193	Present study
C7-O	Delaware	Clinical isolate, sputum	Outbreak	8/10/1994	1	38.34	3,359,446	2970	0	CP015938/ SRR3613194	Present study
C8-S	Colorado	Clinical isolate, sputum	Sporadic	3/12/1993	2	38.31	3,419,998	3077	1 (58,092/38.2)	CP015939/ CP015940/ SRR3613195	Present study; Plasmid named here pCCO, highly similar to pLPL, possible CRISPR
C9-S	Indiana	Clinical isolate, lung	Sporadic	12/30/1982	3	38.30	3,397,139	3114	2 (33,359/39.6; 73,576/39.0)	CP015941/ CP015942/ CP015943/ SRR3613196	Present study; Smaller plasmid named here pCIN1, larger plasmid named pCIN2
C10-S	Nebraska	Clinical isolate, PF	Sporadic	6/7/1990	1	38.33	3,368,319	2975	0	CP015944/ SRR3613197	Present study
C11-O	Georgia	Clinical isolate, sputum	Outbreak	3/18/2009	1	38.29	3,416,875	3025	0	CP015945/ SRR3613173	Present study; Additional outbreak pair 1; Present study
E1-P	California	Environmental isolate	Potential Outbreak	8/14/2013	1	38.34	3,411,341	3023	0	CP015946/ SRR3613174	Present study
E2-N	Nevada	Environmental isolate	Non-outbreak	8/12/2012	2	38.32	3,336,955	3044	1 (71,453/39.09)	CP015947/ CP015948/ SRR3613175	Present study; Plasmid named here pENV
E3-N	Texas	Environmental isolate	Non-outbreak	4/3/2011	1	38.33	3,465,352	3072	0	CP015949/ SRR3613176	Present study
E4-N	Alabama	Environmental isolate	Non-outbreak	8/15/2012	1	38.33	3,416,757	3015	0	CP015950/ SRR3613177	Present study

E5-N	Arkansas	Environmental isolate	Non-outbreak	8/3/2011	2	38.30	3,419,816	3101	1 (73,576/39.03)	CP015951/ CP015952/ SRR3613178	Present study; Plasmid same as pCIN2
E6-N	New Jersey	Environmental isolate	Non-outbreak	4/6/2011	1	38.31	3,405,173	3005	0	CP015953/ SRR3613179	Present study
E7-O	Georgia	Environmental isolate	Outbreak	5/1/2009	1	38.34	3,425,962	3032	0	CP015954/ SRR3613180	Present study; Additional outbreak pair 1
E8-O	Texas	Environmental isolate	Outbreak	6/20/2006	1	38.34	3,455,562	3063	1* /35,481/40.7	CP015955/ SRR3613181	Present study; Additional outbreak pair 2; plasmid named here pO35TX
E9-O	Illinois	Environmental isolate	Outbreak	8/31/2012	2	38.32	3,367,202	3028	1 (48,354/38.39)	CP015956/ CP015957/ SRR3613182	Present study; plasmid named here pOIL
E10-P	Ohio	Environmental isolate	Potential Outbreak	2/23/2007	1	38.32	3,415,557	3020	0	CP015925/ SRR3613184	Present study; Potential outbreak pair
E11-U	Iowa	Environmental isolate	Unknown	10/4/2006	1	38.31	3,402,052	3032	0	CP015926/ SRR3613185	Present study

Footnotes: 1. *, Potential mobilizable, self-replicating plasmid may appear in both integrated and extrachromosomal forms

2. PF, pleural fluid; TTA, trans-tracheal aspirate; BL, bronchial alveolar lavage
